# Supplementary material for: Tuberculosis care quality in urban Nigeria: A cross-sectional study of adherence to screening and treatment initiation guidelines in multi-cadre networks of private health service providers
Source: PLOS Glob Public Health. 2022 Jan 6;2(1):e0000150. doi: 10.1371/journal.pgph.0000150 (PMC10021846; doi:10.1371/journal.pgph.0000150)
Supplement: S1 Annex — (DOCX) [file pgph.0000150.s001.docx]

# **S1: Additional information on sampling, methods, and data collection**

## **Sample frame and stratification**

The study team used multiple data sources to construct the sampling frames for each type of provider. For private facilities, we obtained network facility lists compiled and maintained by SHOPS Plus program staff. For public facilities, the study team first downloaded a list of operational health facilities operating in the same local government areas (LGAs) as SHOPS Plus facilities from the Nigeria Health Facility Registry (NHFR, <http://hfr.health.gov.ng/facilities/hospitals-list>). To identify state-designated facilities offering TB treatment according to directly-observed treatment short course (DOTS) protocols, the study team cross-referenced the NHFR with lists of DOTS facilities directly obtained from and maintained by each state’s TB control program. Facilities owned and operated by Nigeria’s Federal Ministry of Health, armed forces, or not otherwise accessible to the public (i.e., publicly-run facilities for students or public servants only) were excluded from the sampling frame.

The sample frame was then grouped into two strata: private facilities in the SHOPS Plus network and public (government-owned) facilities. Within these two strata, we further defined substrata based on the type of provider. For public facilities, substrata were based on whether the facility was a designated DOTS provider, although all public facilities—regardless of DOTS status—should have the capacity to screen and facilitate diagnosis of TB. These strata and substrata were considered separately for Lagos and Kano. Thus, in total there were 12 strata/substrata groupings (4 substrata for private facilities in the SHOPS Plus network in Kano, 4 substrata for private facilities in the SHOPS Plus network in Lagos, 2 substrata for public facilities in Kano, 2 substrata for public facilities in Lagos).

## **Sample size and power calculations**

We used the hypergeometric sample size to determine a representative number of facilities needed:

𝑛 = (𝑁𝑧^2 𝑝𝑞) / ((MOE^2 (𝑁−1)+𝑧^2 𝑝𝑞))

Where:

n is the number of facilities sampled,

N is the total number of health facilities in a substrata,

p is the probability of an outcome (e.g., a provider at a health facility follows the appropriate protocol for screening a presumptive TB patient),

q = 1 – p,

MOE is the margin of error, and

Z^2 is the confidence level (based on the normal distribution).

The calculation was run for each substrata to ensure that the sample was representative at the appropriate statistical level for all substrata. We used a 95% level of confidence (α = 0.05) for all calculations. Resource limitations and programmatic priorities were considered when determining the margin of error (MOE) for each strata. The assumptions employed in the calculations were based on the strata in question:

1. For private facilities in the SHOPS Plus network, we assumed p = 0.5 (to maximize the variance and thus have the largest potential sample size given other assumptions), and the MOE is ±5%.
2. For public facilities, we assumed p = 0.5 (to maximize the variance and thus have the largest potential sample size given other assumptions), and the MOE is ±10%.

## **Sample selection and verification**

Following sample size calculations, facilities were selected at random within each substratum.

- Each facility in the sampling frame was first assigned a random number (Sample_Num) between 0 and 1 using Excel’s =Rand() function. These random numbers were then sorted smallest to largest.
- A second sample number (Sample_Num2), was created and assigned to facilities in the sampling frame. The facility with the lowest random number is assigned a 1; the facility with the largest random number is assigned N (maximum number of records in the sampling frame). (e.g., in a sampling frame of 50 facilities, records would be numbered 1 to 50).
- For each sampling frame, facilities 1 to X are assigned as the primary sample, with X as the maximum number of facilities calculated for a given strata.
- For each sampling frame, facilities with sample numbers that exceed the sample threshold were assigned as a back-up sample. If there were enough facilities in the sampling frame, the back-up sample was the same size as the primary sample.

Prior to data collection, data collectors conducted a verification exercise on the sampled facilities to confirm each facility’s location and operational status, which is essentially to ensure standardized patients (SPs) could approach facilities inconspicuously. A total of 869 facilities were verified in Lagos and 548 facilities were verified in in Kano. If a facility’s location was unable to be verified, a replacement facility from the back-up sample was randomly selected and verified. The total number of facilities verified exceeded the survey sample to ensure there enough facilities to conduct dry runs and pilot testing.

## **Defining criteria for correct management of SPs**

The criteria for “correct management” were determined by referencing national and international guidelines for management of presumptive TB patients and accounting for the way in which the SHOPS Plus TB program was implemented (i.e., what providers were being trained and told to do). We also considered thresholds set by other, similarly-designed SP studies implemented in other contexts.[1-6] In all cases, TB studies have defined the “correct” threshold leniently (i.e., rather than having to incorporate ALL elements of ideal TB screening, providers just must meet the most important ones). We did the same in our study as well.

### **Correct management for Case 1 - presumptive case scenario**

According to Nigeria’s National TB and Leprosy Control Program (NTBLCP) 2015 guidelines, the main symptom of presumptive pulmonary TB is a productive cough of two weeks, and may include shortness of breath, chest pain, or coughing up blood. Additional symptoms include fever, loss of appetite, weight loss, night sweats, and malaise/tiredness. Because these guidelines (as well as WHO guidelines) are unclear about whether providers must ask about all symptoms to screen we have chosen to set the correct threshold to include inquiry of the “main” TB symptoms specified by the guidelines. We applied the NTBLCP’s 2015 guidelines because these were the reference guidelines in use at the time SHOPS Plus providers were trained in 2018. The criteria for “correct management” for non-clinical cadres (i.e., drug shops, pharmacies, and private stand-alone labs) for this case were like those for clinical providers. The exception is that there is a lower threshold for screening.

The reason for this lower threshold is that shortly after implementation of the SHOPS Plus program began in 2018, it became evident that that non-clinical cadre did not show as much willingness and/or skill to screen as thoroughly for TB as clinical providers. Since all presumptive patients are re-screened and tested by clinical providers before they ever commence TB treatment, the program determined that it was not necessary for non-clinical cadre to screen to the same extent as clinical providers, and that screening could be simplified to one question only: whether patients had been coughing for two weeks or longer.

To be considered to have correctly managed a SP, providers needed to have also recommended or attempted to take an appropriate diagnostic (an x-ray, or a sputum sample for GeneXpert or acid-fast bacilli (AFB) testing) or specifically told the SP that they needed a sputum test or x-ray and would need to go elsewhere for this test (i.e., a referral).

In addition to appropriate screening and recommendations for appropriate diagnostics, providers would have also needed to refrain from prescribing any broad- or narrow-spectrum antibiotics (including anti-TB drugs and fluoroquinolones) and steroids. These medicines can either mask the symptoms of TB, are unnecessary, or inappropriate to dispense in absence of a confirmed TB diagnosis.

### **Correct management for Case 2 – treatment initiation scenario**

As with Case 1, the criteria for “correct management” were determined by referencing NTBLCP and international guidelines for counseling and initiating treatment on confirmed TB patients, consulting with clinical providers familiar with counseling and treatment of TB patients, and considering what SPs were instructed to do in their roles in this case. For example, in Case 2 SPs brought a GeneXpert result into the clinical facility with them indicating they were positive for DS-TB and negative for HIV. Although it is typically recommended that providers confirm positive patients’ HIV status, we did not include it as a criteria for correct management, since it is reasonable that a provider may choose not to insist on HIV confirmation upon seeing the HIV status on the GeneXpert report. Overall, we defined five different criteria elements for providers to demonstrate “correct” treatment initiation.

First, providers needed to confirm the SP’s diagnosis by looking at the GeneXpert result and verbally affirming to the SP that they had TB. Next, providers needed to provide counseling to sufficiently explain TB and its treatment. NTBLCP guidelines specify nine different elements of TB counseling; in this study providers were expected to demonstrate a streamlined set of counseling elements including explaining TB is curable, providing a brief overview of the medicines used to treat TB, describing the duration of treatment (6 months), and emphasizing the importance of taking TB medicine daily. After counseling providers should request the patient to identify a treatment supporter, and initiate TB treatment.

Although NTBLCP guidelines encourage community-based DOTS with a treatment supporter, clinical providers have the option of offering clinic-based DOTS if the client cannot identify a treatment supporter and/or the clinician has doubts that a patient’s treatment supporter can appropriately monitor daily adherence to the TB treatment protocol. As a result, we count three different treatment scenarios as “correct”, since we cannot know with certainty what DOTS scenario the provider determined was best for the SP in this scenario: 1) giving a prescription for TB medications or providing TB drugs directly to the patient to take home; 2) requesting the patient begin observed treatment during the counseling session, or 3) requesting the patient come back later with the treatment supporter to begin observed treatment. Although some providers requested/insisted that SPs began observed treatment during the counseling session, SPs were not able to ingest TB drugs on ethical grounds, so they were trained to tactfully delay initiation and refuse ingesting medications if this circumstance arose. In all treatment initiation instances, providers were also expected to refrain from providing additional, unnecessary medications including non-TB antibiotics and steroids.

### **Details on SP Training and Data Collection**

Executing an SP approach involves covertly sending trained data collectors to a healthcare facility where they present an opening statement for a given scenario, which in our study was either presumptive or recently-confirmed TB. To ensure consistency across SPs and capture providers’ actions we referenced and customized publicly-available manuals and tools to prepare SPs for field deployment.[7-8]

As explained by others,[9] ensuring that SPs presented and responded to providers in a believable and undetectable manner is essential for capturing valid data and requires extensive data collector training. In our study, a team of locally-recruited SPs (19 in Lagos and 12 in Kano) and their supervisors (9 in Lagos and 7 in Kano) were trained over a three-week (144 hour) training and piloting period. The training covered extensive discussions on the SP cases and exit survey tool, and included enactments, role plays, and dry runs in a sample of 94 pilot facilities (62 in Lagos, 32 in Kano). The training also emphasized protocols and techniques to protect SPs from harm (i.e., avoiding invasive procedures such as blood draws, administration of medicines, etc.).

All sampled facilities received an unannounced visit from an SP implementing the Case 1 (the presumptive patient scenario); SHOPS Plus clinical facilities and public DOTS facilities received an additional unannounced visit from an SP implementing Case 2 (the confirmed patient scenario). To prevent detection, different SPs were assigned to implement Case 1 and Case 2; during analysis we verified that facilities were never visited by the same SP. In clinical facilities SPs presented at the main registration point for outpatients and presented their cases to whichever consultant was assigned to attend to them. In non-clinical settings SPs presented their case to whomever was working at the reception desk or retail counter at the time of their visit. Outside of their opening statement (see Table 1 in the main article) SPs were instructed not share any further details (such as additional symptoms, duration of the cough or fever, previous TB, etc.) unless they were directly asked by the provider to provide this information. SPs paid any applicable registration and consultation fees and purchased any medications prescribed. SPs made up to three visit attempts in each sampled facility between 10 June and 12 July 2019.

Directly after each interaction SPs were debriefed by field supervisor to record details of their interaction in a structured exit survey instrument. Field supervisors also collected any items obtained during their interaction (sputum cups, prescription slips, or purchased medicines). A team of trained pharmacists were responsible for identifying and labeling all medicines dispensed to SPs and subsequently categorizing into six categories: palliatives or over-the-counter (i.e., paracetamol or cough syrup); DS-TB medications (rifampicin, isoniazid, ethambutol, pyrazinamide); fluoroquinolones; other antibiotics; steroids; and unlabeled drugs.

## S1 References

1. Das J, Kwan A, Daniels B, et al. Use of standardized patients to assess quality of tuberculosis care: a pilot, cross-sectional study. *The Lancet* 2015;15: 1305-13. doi:10.1016/S1473-3099(15)00077-8.
2. Sylvia S, Xue H, Zhou C, et al. Tuberculosis detection and the challenges of integrated care in rural china: a cross-sectional standardized patient study. *PLoS Med* *2017;* 14(10). doi: [10.1371/journal.pmed.1002405](https://doi.org/10.1371/journal.pmed.1002405).
3. Daniels B, Dolinger A, Bedoya G, et al. Use of standardised patients to assess quality of healthcare in Nairobi, Kenya: a pilot, cross-sectional study with international comparisons. *BMJ Glob Health* 2017*.* 2(2). doi: [10.1136/bmjgh-2017-000333](https://doi.org/10.1136/bmjgh-2017-000333)
4. Miller R and Goodman C. Do chain pharmacies perform better than independent pharmacies? Evidence from a standardised patient study of the management of childhood diarrhoea and suspected tuberculosis in urban India. *BMJ Global Health* 2017, 2(3). doi: 10.1136/bmjgh-2017-000457.
5. Christian CS, Gerdthan UG, Hompashe D, et al. Measuring quality gaps in TB screening in South Africa using standardised patient analysis*. Int J Environ Res Public Health* 2018*;* 15(4): 729. doi:[10.3390/ijerph15040729](https://dx.doi.org/10.3390%2Fijerph15040729).
6. Kwan A, Daniels B, Saria V, et al. Variations in the quality of tuberculosis care in urban India: A cross-sectional, standardized patient study in two cities. *PLoS Med* 2018 Sep 25;15(9):e1002653. doi: 10.1371/journal.pmed.1002653.
7. Chan B, Das V, Mohanan M, et al. (2012) Standardized patients and the measurement of healthcare quality: field guide, manual, and sample instruments. Medical advice, quality, and availability in rural India (MAQARI) Project.
8. Kwan A, Daniels B, Bergkvist S, et al. *Use of standardised patients to measure health care quality: a manual and toolkit for projects in low- and middle-income countries*.  QuTUB Project, McGill University, 2019. <https://www.qutubproject.org/resources>. Date accessed: 2 February 2019.
9. Kwan A, Daniels B, Bergkvist S, et al. Use of standardised patients for healthcare quality research in low- and middle-income countries. *BMJ Global Health* 2019. doi: [10.1136/bmjgh-2019-001669](http://dx.doi.org/10.1136/bmjgh-2019-001669).
