# Supplementary material for: Tuberculosis care quality in urban Nigeria: A cross-sectional study of adherence to screening and treatment initiation guidelines in multi-cadre networks of private health service providers
Source: PLOS Glob Public Health. 2022 Jan 6;2(1):e0000150. doi: 10.1371/journal.pgph.0000150 (PMC10021846; doi:10.1371/journal.pgph.0000150)
Supplement: S2 Annex — (DOCX) [file pgph.0000150.s002.docx]

# S2: Response rates

Out of 1,110 expected Case 1 attempts, SPs were unable to complete 85 (7.7%) interactions. Out of 424 Case 2 attempts, SPs were unable to complete 77 (18.2%) interactions (Table A and B below). There were several reasons SPs could not complete interactions, including a facility being closed after multiple attempts (n=48 in Case 1 and n=16 in Case 2), inability to locate the facility after multiple attempts (n=12 in Case 1 only), absence of a provider who was qualified to serve the SP (n= 20 in Case 1, n=11 in Case 2). In a small number of attempts SPs unexpectedly interacted with an individual identifying as a SHOPS Plus staff member (n=1 in the Case 1, and n=5 in Case 2) or the SP noted that the provider had regarded them with suspicion or accused them faking their illness to obtain drugs on the black market (n=1 in Case 1, and n=4 in Case 2). In Case 2 providers from 37 facilities opted to repeat the sputum test with the SP before initiating treatment. After consulting with program staff and stakeholders, we chose to code these interactions as incomplete and omit them from the analysis, since it is not unreasonable nor explicitly in against protocol for a provider to want to confirm a test result for a new patient who received TB diagnostics from an unfamiliar referral source (which in this scenario was an unnamed pharmacy). Finally, to replace some of the facilities that SPs could not visit due to prolonged closure, SPs visited 6 additional public non-DOTS and 11 additional public DOTS facilities that than originally expected. These additional facilities were unused randomly-selected facilities from the pilot list.

## S2 Table A: Case 1 response rates

|  | Private Drug Shop (All) | | Private Pharm  (All) | | Private Clinical (All) | | Private Labs (All) | | Public Non-DOTS (All) | | Public - DOTS (All) | |
| --- | --- | --- | --- | --- | --- | --- | --- | --- | --- | --- | --- | --- |
|  | N | % | N | % | N | % | N | % | N | % | N | % |
| Number of expected SP client approaches to providers | 389 |  | 120 |  | 283 |  | 111 |  | 66 |  | 141 |  |
| Not included in SP analyses |  |  |  |  |  |  |  |  |  |  |  |  |
| Facility closed after multiple attempts | 24 | 6.2% | 2 | 1.7% | 8 | 2.8% | 3 | 2.7% | 7 | 10.6% | 4 | 2.8% |
| Could not find facility | 6 | 1.5% | 1 | 0.8% | 5 | 1.8% | - | 0.0% | - | 0.0% | - | 0.0% |
| Qualified provider not on site to serve SP | 1 | 0.3% | 2 | 1.7% | 8 | 2.8% | 4 | 3.6% | 1 | 1.5% | 4 | 2.8% |
| Provider acted suspiciously or accused SP of being fake | 1 | 0.3% | - | 0.0% | - | 0.0% | - | 0.0% | - | 0.0% | - | 0.0% |
| SP interacted with SHOPS Plus personnel | - | 0.0% | - | 0.0% | 1 | 0.4% | - | 0.0% | - | 0.0% | - | 0.0% |
| Facility does not treat TB | - | 0.0% | - | 0.0% | - | 0.0% | 1 | 0.9% | - | 0.0% | 2 | 1.4% |
| Other | - | 0.0% | - | 0.0% | - | 0.0% | - | 0.0% | - | 0.0% | - | 0.0% |
| Total not included in analysis | 32 | 8.2% | 5 | 4.2% | 22 | 7.8% | 8 | 7.2% | 8 | 12.1% | 10 | 7.1% |
| Excess facilities used in analysis | - |  | - |  | - |  | - |  | 6 | - | 11 | - |
| Total included in analysis | 355 | 91.3% | 113 | 94.2% | 269 | 95.1% | 100 | 90.1% | 64 | 88.9% | 142 | 100.7% |

## S2 Table B: Case 2 response rates

|  | Private Clinical (All) | | Public DOTS (All) | |
| --- | --- | --- | --- | --- |
|  | N | % | N | % |
| Number of SP expected client approaches to providers | 283 |  | 141 |  |
| Not included in SP analyses |  |  |  |  |
| Provider requests repeat sputum test before initiating treatment | 27 | 9.5% | 10 | 7.1% |
| Qualified provider not on site to serve SP | 7 | 2.5% | 4 | 2.8% |
| SP interacted with SHOPS Plus personnel | 5 | 1.8% | - | 0.0% |
| Provider acted suspiciously or accused SP of being fake | 4 | 1.4% | - | 0.0% |
| Facility closed after multiple attempts | 1 | 0.4% | 15 | 10.6% |
| Facility does not treat TB | 2 | 0.7% | 2 | 1.4% |
| Could not find facility | - | 0.0% | - | 0.0% |
| Other | - | 0.0% | - | 0.0% |
| Total not included in analysis | 46 | 16.3% | 31 | 22.0% |
| Total included in analysis | 228 | 83.7% | 119 | 78.0% |
