# Supplementary material for: Tuberculosis care quality in urban Nigeria: A cross-sectional study of adherence to screening and treatment initiation guidelines in multi-cadre networks of private health service providers
Source: PLOS Glob Public Health. 2022 Jan 6;2(1):e0000150. doi: 10.1371/journal.pgph.0000150 (PMC10021846; doi:10.1371/journal.pgph.0000150)
Supplement: S3 Annex — (DOCX) [file pgph.0000150.s003.docx]

# **S3 file: Case 1 detailed results tables - including state-specific results**

The tables below provide detailed results for Case 1 (presumptive patient scenario). Table A presents for all private (SHOPS Plus) facilities that were visited by SPs for this study. Table B shows Lagos-specific results and Table C shows Kano-specific results. Tables D-G show results from a sensitivity analysis we conducted where the criterion related to prescriptions was narrowed so that it would only apply to prescriptions of steroids and fluroquinolones (and not other broad- and narrow-spectrum antibiotics). Tables I and J show correlation analysis results for private SHOPS Plus and Public facilities. Table K shows full regression analysis results for Case 1. Table L shows Case 1 results for all private SHOPS Plus facilities benchmarked to all public facilities. Table D shows results for all public (overall and for each state, by DOTS and non-DOTS status).

## Table A: Case 1– all results

| SHOPS Plus Facilities - Case 1: Presumptive Patient Scenario | SHOPS Plus Drug Shops  (All, n=355) | | SHOPS Plus Pharmacies  (All, n=113) | | SHOPS Plus Laboratories  (All, n=100) | | SHOPS Plus Clinical  (All, n=269) | |
| --- | --- | --- | --- | --- | --- | --- | --- | --- |
|  | % | CI | % | CI | % | CI | % | CI |
| Highest qualification of provider seen |  |  |  |  |  |  |  |  |
| Consultant | 0.80% | (0.4%, 1.9%) | 0.90% | (0.3%, 2.2%) | 4.0% | (2.8%, 5.7%) | 90.70% | (88.2%, 92.7%) |
| Nurse | 1.10% | (0.6%, 2.3%) | 0% | (.%, .%) | 0.0% | (.%, .%) | 7.80% | (6%, 10.1%) |
| Pharmacist/Drug Shops | 81.70% | (78.7%, 84.4%) | 75.20% | (71.6%, 78.5%) | 0.0% | (.%, .%) | 0% | (.%, .%) |
| Shop assistant | 13% | (10.7%, 15.6%) | 18.60% | (15.7%, 21.8%) | 0.0% | (.%, .%) | 0% | (.%, .%) |
| Registrar/receptionist | 0.30% | (0.1%, 1.2%) | 0.90% | (0.3%, 2.2%) | 17.0% | (14.2%, 20.2%) | 0% | (.%, .%) |
| Laboratory technician | 0% | (.%, .%) | 0% | (.%, .%) | 76.0% | (72.5%, 79.2%) | 0% | (.%, .%) |
| Other | 0.80% | (0.4%, 1.9%) | 1.80% | (0.9%, 3.4%) | 1.0% | (.5%, 2.1%) | 1.50% | (.7%, 2.9%) |
| Don't know | 2.30% | (1.4%, 3.7%) | 2.70% | (1.6%, 4.5%) | 2.0% | (1.1%, 3.6%) | 0% | (.%, .%) |
| Gender of provider (highest cadre seen) |  |  |  |  |  |  |  |  |
| Female | 27.90% | (25%, 31%) | 49.60% | (45.5%, 53.6%) | 44.0% | (40.2%, 47.9%) | 27.50% | (24.2%, 31.1%) |
| Male | 72.10% | (69%, 75%) | 50.40% | (46.4%, 54.5%) | 56.0% | (52.1%, 59.8%) | 72.50% | (68.9%, 75.8%) |
| Provider asked about duration of cough (Drug Shops / Pharmacies) | 70.40% | (67.1%, 73.6%) | 64.60% | (60.5%, 68.5%) | 88.0% | (85.2%, 90.4%) |  |  |
| Provider confirmed duration of productive cough and one more symptom (Clinical) |  |  |  |  |  |  | 72.90% | (69.5%, 76%) |
| Provider took sputum sample, rec'd chest X-ray, or referred SP for sputum test | 33.80% | (30.4%, 37.4%) | 15.90% | (13.3%, 19%) | 93.0% | (90.7%, 94.8%) | 55.80% | (51.9%, 59.5%) |
| No unnecessary sale of antibiotics, anti-TB drugs, or steroids | 44.80% | (41.2%, 48.5%) | 37.20% | (33.3%, 41.2%) | 100.0% | (.%, .%) | 60.60% | (56.8%, 64.3%) |
| Correct Management (met all 3 criteria) | 21.40% | (18.5%, 24.6%) | 8% | (6%, 10.4%) | 83.0% | (79.8%, 85.8%) | 31.20% | (27.7%, 34.9%) |
| Met 1 criteria | 37.50% | (34%, 41.1%) | 44.20% | (40.3%, 48.3%) | 0.02 | (1.1%, 3.6%) | 24.90% | (21.8%, 28.2%) |
| Provider asked about duration of cough (Drug Shops / Pharmacies) | 79.70% | (74.4%, 84.1%) | 78% | (72.5%, 82.6%) | 0 | (.%, .%) |  |  |
| Provider confirmed duration of productive cough and one more symptom (Clinical) |  |  |  |  |  |  | 44.80% | (37.6%, 52.2%) |
| Provider took sputum sample, rec'd chest X-ray, or referred SP for sputum test | 3% | (1.5%, 5.9%) | 0% | (.%, .%) | 0 | (.%, .%) | 10.40% | (6.8%, 15.8%) |
| No unnecessary sale of antibiotics, anti-TB drugs, or steroids | 17.30% | (13.2%, 22.4%) | 22% | (17.4%, 27.5%) | 1 | (.%, .%) | 44.80% | (37.8%, 52%) |
| Met 2 criteria | 23.70% | (20.7%, 26.9%) | 24.80% | (21.5%, 28.4%) | 0.15 | (12.4%, 18%) | 35.30% | (31.7%, 39.1%) |
| Duration/productive cough and sputum sample | 28.60% | (22.3%, 35.9%) | 21.40% | (15.8%, 28.4%) | 0 | (.%, .%) | 48.40% | (42%, 54.9%) |
| Sputum sample and no unnecessary sale | 19% | (13.8%, 25.7%) | 10.70% | (6.8%, 16.5%) | 0.667 | (56.5%, 75.5%) | 13.70% | (9.7%, 19%) |
| Duration/productive cough and no unnecessary sale | 52.40% | (44.9%, 59.8%) | 67.90% | (60.2%, 74.7%) | 33.3% | (24.5%, 43.5%) | 37.90% | (31.7%, 44.5%) |
| Met no criteria | 17.40% | (14.9%, 20.4%) | 23.00% | (19.6%, 26.8%) | 0.00% | NA | 8.60% | (6.4%, 10.5%) |
| Met at least one criteria | 82.60% | (79.6%, 85.1%) | 77.00% | (73.2%, 80.4%) | 100.00% | NA | 91.40% | (89.5%, 93.6%) |
| CI: 95% confidence interval | | | | | | | | |

## Table B: Case 1– Lagos state results

| SHOPS Plus Facilities - Case 1: Presumptive Patient Scenario | SHOPS Plus Drug Shops (Lagos, n=171) | | SHOPS Plus Pharmacies  (Lagos, n=94) | | SHOPS Plus Laboratories (Lagos, n=80) | | SHOPS Plus Clinical  (Lagos, n=196) | |
| --- | --- | --- | --- | --- | --- | --- | --- | --- |
|  | % | CI | % | CI | % | CI | % | CI |
| Highest qualification of provider seen |  |  |  |  |  |  |  |  |
| Consultant | 1.80% | (0.8%, 4%) | 1.10% | (0.4%, 2.7%) | 5% | (3.5%, 7.2%) | 90.30% | (87%, 92.9%) |
| Nurse | 2.30% | (1.1%, 4.7%) | 0% | (.%, .%) | 0% | (.%, .%) | 7.70% | (5.4%, 10.7%) |
| Pharmacist/PPMV | 77.80% | (72.9%, 82%) | 76.60% | (72.3%, 80.4%) | 0% | (.%, .%) | 0% | (.%, .%) |
| Shop assistant | 11.10% | (8.1%, 15.1%) | 16% | (12.7%, 19.8%) | 0% | (.%, .%) | 0% | (.%, .%) |
| Registrar/receptionist | 0.60% | (0.1%, 2.4%) | 1.10% | (0.4%, 2.7%) | 16.20% | (13.4%, 19.6%) | 0% | (.%, .%) |
| Laboratory technician | 0% | (.%, .%) | 0% | (.%, .%) | 76.20% | (72.5%, 79.6%) | 0% | (.%, .%) |
| Other | 1.80% | (0.8%, 4%) | 2.10% | (1.1%, 4.1%) | 1.30% | (.6%, 2.6%) | 2% | (1%, 4%) |
| Don't know | 4.70% | (2.8%, 7.6%) | 3.20% | (1.9%, 5.4%) | 1.30% | (.6%, 2.6%) | 0% | (.%, .%) |
| Gender of provider (highest cadre seen) |  |  |  |  |  |  |  |  |
| Female | 48.50% | (43.1%, 54%) | 53.20% | (48.4%, 58%) | 47.50% | (43.4%, 51.7%) | 31.10% | (26.7%, 35.9%) |
| Male | 51.50% | (46%, 56.9%) | 46.80% | (42%, 51.6%) | 52.50% | (48.3%, 56.6%) | 68.90% | (64.1%, 73.3%) |
| Provider asked about duration of cough (Drug Shops / Pharmacies) | 57.30% | (51.8%, 62.7%) | 60.60% | (55.8%, 65.2%) | 90% | (87.2%, 92.2%) |  |  |
| Provider confirmed duration of productive cough and one more symptom (Clinical) |  |  |  |  |  |  | 77.60% | (73.1%, 81.4%) |
| Provider took sputum sample, rec'd chest X-ray, or referred SP for sputum test | 31.60% | (26.7%, 36.9%) | 13.80% | (10.8%, 17.5%) | 93.80% | (91.4%, 95.5%) | 58.20% | (53.2%, 63%) |
| No unnecessary sale of antibiotics, anti-TB drugs, or steroids | 40.40% | (35.1%, 45.9%) | 36.20% | (31.7%, 40.9%) | 100% | (.%, .%) | 62.80% | (57.9%, 67.4%) |
| Correct Management (met all 3 criteria) | 19.30% | (15.3%, 24%) | 7.40% | (5.3%, 10.4%) | 85% | (81.8%, 87.7%) | 34.70% | (30.1%, 39.5%) |
| Met 1 criteria | 35.10% | (30%, 40.5%) | 41.50% | (36.8%, 46.3%) | 1.30% | (0.6%, 2.6%) | 20.90% | (17.2%, 25.2%) |
| Provider asked about duration of cough (Drug Shops / Pharmacies) | 71.70% | (62.6%, 79.3%) | 76.90% | (70%, 82.6%) | 0% | (.%, .%) |  |  |
| Provider confirmed duration of productive cough and one more symptom (Clinical) |  |  |  |  |  |  | 56.10% | (45.2%, 66.4%) |
| Provider took sputum sample, rec'd chest X-ray, or referred SP for sputum test | 3.30% | (1.2%, 8.9%) | 0% | (.%, .%) | 0% | (.%, .%) | 9.80% | (5%, 18.3%) |
| No unnecessary sale of antibiotics, anti-TB drugs, or steroids | 25% | (17.8%, 33.9%) | 23.10% | (17.4%, 30%) | 100% | (.%, .%) | 34.10% | (24.7%, 45%) |
| Met 2 criteria | 18.10% | (14.3%, 22.8%) | 23.40% | (19.6%, 27.7%) | 13.80% | (11.1%, 16.9%) | 36.70% | (32.1%, 41.6%) |
| Duration/productive cough and sputum sample | 32.30% | (21.5%, 45.3%) | 18.20% | (11.7%, 27.1%) | 0% | (.%, .%) | 43.10% | (35.2%, 51.3%) |
| Sputum sample and no unnecessary sale | 29% | (18.8%, 42%) | 9.10% | (4.8%, 16.7%) | 63.60% | (52.3%, 73.6%) | 15.30% | (10.3%, 22.1%) |
| Duration/productive cough and no unnecessary sale | 38.70% | (27.1%, 51.8%) | 72.70% | (63%, 80.7%) | 36.40% | (26.4%, 47.7%) | 41.70% | (33.9%, 49.9%) |
| Met no criteria | 27.50% | (22.8%, 32.7%) | 27.70% | (23.6%, 32.2%) | -0.10% | NA | 7.70% | (5.0%, 10.2%) |
| Met at least one criteria | 72.50% | (67.3%, 77.2%) | 72.30% | (67.8%, 76.4%) | 100.10% | NA | 92.30% | (89.8%, 95.0%) |
| CI: 95% confidence interval | | | | | | | | |

## Table C: Case 1– Kano state results

| SHOPS Plus Facilities - Case 1: Presumptive Patient Scenario | SHOPS Plus Drug Shops (Kano, n=184) | | SHOPS Plus Pharmacies (Kano, n=19) | | SHOPS Plus Laboratories (Kano, n=20) | | SHOPS Plus Clinical (Kano, n=73) | |
| --- | --- | --- | --- | --- | --- | --- | --- | --- |
|  | % | CI | % | CI | % | CI | % | CI |
| Highest qualification of provider seen |  |  |  |  |  |  |  |  |
| Consultant | 0% | (.%, .%) | 0% | (.%, .%) | 0% | (.%, .%) | 91.80% | (88.6%, 94.1%) |
| Nurse | 0% | (.%, .%) | 0% | (.%, .%) | 0% | (.%, .%) | 8.20% | (5.9%, 11.4%) |
| Pharmacist/Drug Shops | 85.30% | (81.4%, 88.5%) | 68.40% | (63.1%, 73.3%) | 0% | (.%, .%) | 0% | (.%, .%) |
| Shop assistant | 14.70% | (11.5%, 18.6%) | 31.60% | (26.7%, 36.9%) | 0% | (.%, .%) | 0% | (.%, .%) |
| Registrar/receptionist | 0% | (.%, .%) | 0% | (.%, .%) | 20% | (12.7%, 30%) | 0% | (.%, .%) |
| Laboratory technician | 0% | (.%, .%) | 0% | (.%, .%) | 75% | (64.6%, 83.2%) | 0% | (.%, .%) |
| Other | 0% | (.%, .%) | 0% | (.%, .%) | 0% | (.%, .%) | 0% | (.%, .%) |
| Don't know | 0% | (.%, .%) | 0% | (.%, .%) | 5% | (1.9%, 12.4%) | 0% | (.%, .%) |
| Gender of provider (highest cadre seen) |  |  |  |  |  |  |  |  |
| Female | 8.70% | (6.3%, 12%) | 31.60% | (26.7%, 36.9%) | 30% | (21.1%, 40.7%) | 17.80% | (14.3%, 22%) |
| Male | 91.30% | (88%, 93.7%) | 68.40% | (63.1%, 73.3%) | 70% | (59.3%, 78.9%) | 82.20% | (78%, 85.7%) |
| Provider asked about duration of cough (Drug Shops / Pharmacies) | 82.6% | (78.5%, 86.1%) | 84.2% | (79.7%, 87.8%) | 80.0% | (70%, 87.3%) |  |  |
| Provider confirmed duration of productive cough and one more symptom (Clinical) |  |  |  |  |  |  | 60.3% | (55.3%, 65.1%) |
| Provider took sputum sample, rec'd chest X-ray, or referred SP for sputum test | 35.9% | (31.2%, 40.8%) | 26.3% | (21.7%, 31.5%) | 90.0% | (81.4%, 94.9%) | 49.3% | (44.3%, 54.3%) |
| No unnecessary sale of antibiotics, anti-TB drugs, or steroids | 48.9% | (43.9%, 53.9%) | 42.1% | (36.8%, 47.6%) | 100.0% | (.%, .%) | 54.8% | (49.8%, 59.7%) |
| Correct Management (met all 3 criteria) | 23.4% | (19.4%, 27.9%) | 10.5% | (7.6%, 14.4%) | 75.0% | (64.6%, 83.2%) | 21.9% | (18%, 26.3%) |
| Met 1 criteria | 39.70% | (34.9%, 44.7%) | 57.90% | (52.4%, 63.2%) | 5% | (1.9%, 12.4%) | 35.60% | (31%, 40.6%) |
| Provider asked about duration of cough (Drug Shops / Pharmacies) | 86.30% | (79.9%, 90.9%) | 81.80% | (75.5%, 86.8%) | 0% | (.%, .%) |  |  |
| Provider confirmed duration of productive cough and one more symptom (Clinical) |  |  |  |  |  |  | 26.90% | (20.1%, 35%) |
| Provider took sputum sample, rec'd chest X-ray, or referred SP for sputum test | 2.70% | (1.1%, 6.9%) | 0% | (.%, .%) | 0% | (.%, .%) | 11.50% | (7.2%, 18.1%) |
| No unnecessary sale of antibiotics, anti-TB drugs, or steroids | 11% | (6.9%, 17%) | 18.20% | (13.2%, 24.5%) | 100% | (.%, .%) | 61.50% | (53.1%, 69.3%) |
| Met 2 criteria | 28.80% | (24.5%, 33.5%) | 31.60% | (26.7%, 36.9%) | 20% | (12.7%, 30%) | 31.50% | (27%, 36.3%) |
| Duration/productive cough and sputum sample | 26.40% | (19%, 35.4%) | 33.30% | (24.8%, 43.2%) | 0% | (.%, .%) | 65.20% | (56.3%, 73.2%) |
| Sputum sample and no unnecessary sale | 13.20% | (8.1%, 20.9%) | 16.70% | (10.5%, 25.3%) | 75% | (49.6%, 90.1%) | 8.70% | (4.8%, 15.2%) |
| Duration/productive cough and no unnecessary sale | 60.40% | (51%, 69%) | 50% | (40.3%, 59.7%) | 25% | (9.9%, 50.4%) | 26.10% | (19%, 34.7%) |
| Met no criteria | 8.10% | (5.8%, 11.3%) | 0.00% | NA | 0.00% | NA | 11.00% | (8.2%, 14.5%) |
| Met at least one criteria | 91.90% | (88.7%, 94.2%) | 100.00% | NA | 100.00% | NA | 89.00% | (85.5%, 91.8%) |
| CI: 95% confidence interval | | | | | | | | |

## Table D: Sensitivity Analysis w/ relaxed Rx criterion- Case 1 All SHOPS Plus

| SHOPS Plus Facilities - Presumptive Patient Scenario | SHOPS+ Drug Shops (All, n=355) | | SHOPS+ Pharmacies (All, n=113) | | SHOPS+ Labs (All, n=100) | | SHOPS+ Clinical (All, n=269) | |
| --- | --- | --- | --- | --- | --- | --- | --- | --- |
|  | % | CI | % | CI | % | CI | % | CI |
| Provider asked about duration of cough (Drug Shops, Labs, Pharmacies) | 70.40% | (67.1%, 73.6%) | 64.60% | (60.5%, 68.5%) | 88.0% | (85.2%, 90.4%) |  |  |
| Provider confirmed duration of productive cough and one more symptom (Clinical) |  |  |  |  |  |  | 72.90% | (69.5%, 76%) |
| Provider took sputum sample, rec'd chest X-ray, or referred SP for sputum test | 33.80% | (30.4%, 37.4%) | 15.90% | (13.3%, 19%) | 93.0% | (90.7%, 94.8%) | 55.80% | (51.9%, 59.5%) |
| No unnecessary sale of fluoroquinolones, anti-TB drugs, or steroids | 76.90% | (73.7%, 79.8%) | 63.70% | (59.6%, 67.6%) | 100.0% | (.%, .%) | 74.30% | (70.9%, 77.6%) |
| Correct Management (met all 3 criteria) | 26.20% | (23.1%, 29.5%) | 10% | (7.6%, 12.3%) | 83.0% | (79.8%, 85.8%) | 36.80% | (33.2%, 40.6%) |

## Table E: Sensitivity Analysis w/ relaxed Rx criterion- Case 1 Lagos SHOPS Plus

| SHOPS Plus Facilities - Presumptive Patient Scenario | SHOPS+ Drug SHOPS (Lagos, n=171) | | SHOPS+ Pharmacies (Lagos, n=94) | | | SHOPS+ Labs (Lagos, n=80) | | SHOPS+ Clinical (Lagos, n=196) | |
| --- | --- | --- | --- | --- | --- | --- | --- | --- | --- |
|  | % | CI | | % | CI | % | CI | % | CI |
| Provider asked about duration of cough (Drug Shops, Labs, Pharmacies) | 57.30% | (51.8%, 62.7%) | | 60.60% | (55.8%, 65.2%) | 90% | (87.2%, 92.2%) |  |  |
| Provider confirmed duration of productive cough and one more symptom (Clinical) |  |  | |  |  |  |  | 77.60% | (73.1%, 81.4%) |
| Provider took sputum sample, rec'd chest X-ray, or referred SP for sputum test | 31.60% | (26.7%, 36.9%) | | 13.80% | (10.8%, 17.5%) | 93.80% | (91.4%, 95.5%) | 58.20% | (53.2%, 63%) |
| No Rx/sale of fluoroquinolones, anti-TB drugs, or steroids | 66.10% | (60.7%, 71.1%) | | 58.50% | (53.7%, 63.2%) | 100% | (.%, .%) | 74.50% | (69.9%, 78.6%) |
| Correct Management (met all 3 criteria) | 21.10% | (16.9%, 25.9%) | | 8.50% | (6.2%, 11.6%) | 85% | (81.8%, 87.7%) | 38.80% | (34.1%, 43.7%) |

## Table F: Sensitivity Analysis w/ relaxed Rx criterion- Case 1 Kano SHOPS Plus

| SHOPS Plus Facilities - Presumptive Patient Scenario | SHOPS+ Drug Shops (Kano, n=184) | | SHOPS+ Pharmacies (Kano, n=19) | | SHOPS+ Labs (Kano, n=20) | | SHOPS+ Clinical (Kano, n=73) | |
| --- | --- | --- | --- | --- | --- | --- | --- | --- |
|  | % | CI | % | CI | % | CI | % | CI |
| Provider asked about duration of cough (Drug Shops, Labs, Pharmacies) | 82.6% | (78.5%, 86.1%) | 84.2% | (79.7%, 87.8%) | 80.0% | (70%, 87.3%) |  |  |
| Provider confirmed duration of productive cough and one more symptom (Clinical) |  |  |  |  |  |  | 60.3% | (55.3%, 65.1%) |
| Provider took sputum sample, rec'd chest X-ray, or referred SP for sputum test | 35.9% | (31.2%, 40.8%) | 26.3% | (21.7%, 31.5%) | 90.0% | (81.4%, 94.9%) | 49.3% | (44.3%, 54.3%) |
| No Rx/sale of fluoroquinolones, anti-TB drugs, or steroids | 87.0% | (83.2%, 90%) | 89.5% | (85.6%, 92.4%) | 100.0% | (.%, .%) | 74.0% | (69.3%, 78.1%) |
| Correct Management (met all 3 criteria) | 31.0% | (26.6%, 35.8%) | 15.8% | (12.2%, 20.3%) | 75.0% | (64.6%, 83.2%) | 31.5% | (27%, 36.3%) |

## Table G: Sensitivity Analysis w/ relaxed Rx criterion- Case 1 Public Clinical Facilities

| Public Facilities - Presumptive Patient Scenario | | Public Non DOTS  (All, n=64) | | Public DOTS  (All, n=142) | | | Public Non DOTS  (Lagos, n=23) | | | Public DOTS  (Lagos, n= 72) | | | Public Non DOTS  (Kano, n=41) | | | Public DOTS (Kano, n=70)) | | |
| --- | --- | --- | --- | --- | --- | --- | --- | --- | --- | --- | --- | --- | --- | --- | --- | --- | --- | --- |
|  |  | % | CI | % | | CI | % | | CI | % | | CI | % | | CI | % | | CI |
| Provider confirmed duration of productive cough and one more symptom (Clinical) | | 35.9% | (29.1%, 43.5%) | 37.3% | | (30.9%, 44.2%) | 30.4% | | (19.9%, 43.6%) | 45.8% | | (36.2%, 55.8%) | 39.0% | | (30.2%, 48.7%) | 28.6% | | (20.5%, 38.3%) |
| Provider took sputum sample, rec'd chest X-ray, or referred SP for sputum test | | 46.9% | (39.5%, 54.4%) | 51.4% | | (44.4%, 58.3%) | 56.5% | | (43.4%, 68.8%) | 52.8% | | (42.8%, 62.5%) | 41.5% | | (32.4%, 51.1%) | 50.0% | | (40.2%, 59.8%) |
| No Rx/sale of fluoroquinolones, anti-TB drugs, or steroids | | 92.2% | (87.3%, 95.3%) | 83.8% | | (78%, 88.3%) | 82.6% | | (70.5%, 90.4%) | 87.5% | | (79.3%, 92.8%) | 97.6% | | (92%, 99.3%) | 80.0% | | (70.9%, 86.8%) |
| Correct Management (met all 3 criteria) | | 26.6% | (20.4%, 33.8%) | 21.1% | | (16%, 27.4%) | 26.1% | | (16.3%, 39%) | 23.6% | | (16.2%, 33.1%) | 26.8% | | (19.2%, 36.1%) | 18.6% | | (12%, 27.6%) |
| Table H: Case 1 Correlation Tables, by Private Sector Cadre | | | | | | | | | | | | | | | |  |  |  |
|  | | | | Pairwise tetrachoric correlation coefficients | | | | | | | | |  | | |  |  |  |
|  | | | | SHOPS+ Drug Shop (All, n=355) | | | | | | | | | | | |  |  |  |
|  | | | | Duration of cough | | | Sputum sample, chest X-ray, or referred MC for sputum test | | | No unnecessary sale of antibiotics, anti-TB drugs, or steroids | | | Successful completion of scenario | | |  |  |  |
| Duration of cough | | | | 1.00 | | |  | | |  | | |  | | |  |  |  |
| Sputum sample, chest X-ray, or referred SP for sputum test | | | | 0.36 | | | 1.00 | | |  | | |  | | |  |  |  |
| No unnecessary sale of antibiotics, anti-TB drugs, or steroids | | | | 0.17 | | | 0.68 | | | 1.00 | | |  | | |  |  |  |
| Successful completion of scenario | | | | 1.00 | | | 1.00 | | | 1.00 | | | 1.00 | | |  |  |  |
|  | | | |  | | |  | | |  | | |  | | |  |  |  |
|  | | | | SHOPS+ Pharmacy (All, n=113) | | | | | | | | | | | |  |  |  |
|  | | | | Duration of cough | | | Sputum sample, chest X-ray, or referred SP for sputum test | | | No unnecessary sale of antibiotics, anti-TB drugs, or steroids | | | Successful completion of scenario | | |  |  |  |
| Duration of cough | | | | 1.00 | | |  | | |  | | |  | | |  |  |  |
| Sputum sample, chest X-ray, or referred SP for sputum test | | | | 0.36 | | | 1.00 | | |  | | |  | | |  |  |  |
| No unnecessary sale of antibiotics, anti-TB drugs, or steroids | | | | 0.05 | | | 0.47 | | | 1.00 | | |  | | |  |  |  |
| Successful completion of scenario | | | | 1.00 | | | 1.00 | | | 1.00 | | | 1.00 | | |  |  |  |
|  | | | |  | | |  | | |  | | |  | | |  |  |  |
|  | | | | SHOPS+ Labs (All, n=100) | | | | | | | | | | | |  |  |  |
|  | | | | Duration of cough | | | Sputum sample, chest X-ray, or referred SP for sputum test | | | No unnecessary sale of antibiotics, anti-TB drugs, or steroids | | | Successful completion of scenario | | |  |  |  |
| Duration of cough | | | | 1.00 | | |  | | |  | | |  | | |  |  |  |
| Sputum sample, chest X-ray, or referred SP for sputum test | | | | 0.33 | | | 1.00 | | |  | | |  | | |  |  |  |
| No unnecessary sale of antibiotics, anti-TB drugs, or steroids | | | | NA | | | NA | | | NA | | |  | | |  |  |  |
| Successful completion of scenario | | | | 1.00 | | | 1.00 | | | NA | | | 1.00 | | |  |  |  |
|  | | | |  | | |  | | |  | | |  | | |  |  |  |
|  | | | | SHOPS+ Clinical (All, n=269) | | | | | | | | | | | |  |  |  |
|  | | | | Duration of cough | | | Sputum sample, chest X-ray, or referred SP for sputum test | | | No unnecessary sale of antibiotics, anti-TB drugs, or steroids | | | Successful completion of scenario | | |  |  |  |
| Duration of cough | | | | 1.00 | | |  | | |  | | |  | | |  |  |  |
| Sputum sample, chest X-ray, or referred SP for sputum test | | | | 0.55 | | | 1.00 | | |  | | |  | | |  |  |  |
| No unnecessary sale of antibiotics, anti-TB drugs, or steroids | | | | 0.04 | | | 0.15 | | | 1.00 | | |  | | |  |  |  |
| Successful completion of scenario | | | | 1.00 | | | 1.00 | | | 1.00 | | | 1.00 | | |  |  |  |

## Table I: Case 1 Regression Analysis – Individual Case Elements and Overall Correct Management for Private Facilities

|  | **Provider confirmed duration of productive (clinical) cough and one more symptom** | | | | **Provider took sputum sample, rec'd chest X-ray, or referred SP for sputum test** | | | | **No unnecessary sale of antibiotics, anti-TB drugs, or steroids** | | | | **Provider successfully managed SP** | | | |
| --- | --- | --- | --- | --- | --- | --- | --- | --- | --- | --- | --- | --- | --- | --- | --- | --- |
|  | OR | SE | 95% CI | | Odds ratio | SE | 95% CI | | Odds ratio | SE | 95% CI | | Odds ratio | SE | 95% CI | |
|  |  |  | Lower | Upper |  |  | Lower | Upper |  |  | Lower | Upper |  |  | Lower | Upper |
| *Facility class / provider* |  |  |  |  |  |  |  |  |  |  |  |  |  |  |  |  |
| Drug Shop/ Drug SHOP Proprietors | 3.71 | 2.21* | 1.15 | 11.94 | 0.58 | 0.17 | 0.33 | 1.02 | 0.77 | 0.25 | 0.41 | 1.46 | 1.19 | 0.45 | 0.57 | 2.49 |
| Drug Shop/Other Drug Shop Staff | 0.72 | 0.41 | 0.24 | 2.17 | 0.14 | 0.06*** | 0.07 | 0.31 | 0.66 | 0.32 | 0.25 | 1.71 | 0.33 | 0.22 | 0.09 | 1.19 |
| Pharmacy/Pharmacist | 2.79 | 1.78 | 0.8 | 9.75 | 0.45 | 0.3 | 0.12 | 1.66 | 0.48 | 0.41 | 0.09 | 2.61 | 0.51 | 0.46 | 0.09 | 2.95 |
| Pharmacy/Other staff | 0.84 | 0.36 | 0.36 | 1.95 | 0.26 | 0.09*** | 0.13 | 0.52 | 0.73 | 0.3 | 0.32 | 1.64 | 0.02 | 0.02** | 0 | 0.2 |
| Lab/Lab Technician | 3.94 | 0.97*** | 2.43 | 6.39 | 20.59 | 14.06*** | 5.4 | 78.48 | 1 | . |  |  | 18.78 | 7.68*** | 8.43 | 41.86 |
| Lab/Other-staff | 0.78 | 0.43 | 0.26 | 2.29 | 2.48 | 1.16 | 0.99 | 6.21 | 1 | . |  |  | 2.24 | 0.75* | 1.16 | 4.31 |
| Clinical/Non-Consultant | 0.2 | 0.12** | 0.06 | 0.66 | 0.81 | 0.35 | 0.34 | 1.91 | 1.31 | 0.7 | 0.46 | 3.73 | 0.36 | 0.21 | 0.11 | 1.16 |
| Clinical/Consultant (reference) |  |  |  |  |  |  |  |  |  |  |  |  |  |  |  |  |
|  |  |  |  |  |  |  |  |  |  |  |  |  |  |  |  |  |
| *State* |  |  |  |  |  |  |  |  |  |  |  |  |  |  |  |  |
| Lagos | 2.08 | 0.68* | 1.1 | 3.94 | 1.56 | 0.29* | 1.09 | 2.24 | 2.15 | 0.47*** | 1.39 | 3.31 | 2.07 | 0.51** | 1.28 | 3.34 |
| Kano (reference) |  |  |  |  |  |  |  |  |  |  |  |  |  |  |  |  |
| *Gender: Provider/client* |  |  |  |  |  |  |  |  |  |  |  |  |  |  |  |  |
| Female/Female | 1.18 | 0.28 | 0.74 | 1.87 | 1.54 | 0.41 | 0.92 | 2.59 | 0.9 | 0.25 | 0.52 | 1.54 | 2.4 | 0.66** | 1.4 | 4.1 |
| Male/Female | 1.68 | 0.29** | 1.19 | 2.37 | 1.36 | 0.21* | 1.01 | 1.83 | 1.05 | 0.12 | 0.84 | 1.31 | 1.73 | 0.42* | 1.07 | 2.78 |
| Female/Male | 1.11 | 0.51 | 0.45 | 2.74 | 1.87 | 0.45** | 1.17 | 2.98 | 1.1 | 0.25 | 0.7 | 1.72 | 2.93 | 0.94*** | 1.56 | 5.49 |
| Male/Male (reference) |  |  |  |  |  |  |  |  |  |  |  |  |  |  |  |  |
| *Ave. number of patients in waiting room* |  |  |  |  |  |  |  |  |  |  |  |  |  |  |  |  |
| Less than 1 (reference) |  |  |  |  |  |  |  |  |  |  |  |  |  |  |  |  |
| 1 to <2 Patients | 1.85 | 0.8 | 0.79 | 4.33 | 1.02 | 0.23 | 0.66 | 1.58 | 1.17 | 0.32 | 0.68 | 2.01 | 1.11 | 0.32 | 0.64 | 1.95 |
| 2 to <6 Patients | 1.15 | 0.53 | 0.46 | 2.85 | 0.6 | 0.2 | 0.3 | 1.17 | 0.79 | 0.26 | 0.41 | 1.5 | 0.69 | 0.2 | 0.39 | 1.24 |
| 6+ Patients | 0.95 | 0.48 | 0.36 | 2.55 | 0.72 | 0.26 | 0.35 | 1.46 | 0.86 | 0.46 | 0.3 | 2.44 | 1.11 | 0.41 | 0.54 | 2.29 |
| SE: Robust Standard Error; CI: Confidence Interval; * p<0.05; ** p<0.01; *** p<0.001 | | | | | | | | | | | | | | | | |

## Table J: Benchmarking private providers’ SP management to the public sector - Case 1 results in Lagos and Kano

| Provider type | Provider successfully managed SP | | | | | | | | | | | | | | |
| --- | --- | --- | --- | --- | --- | --- | --- | --- | --- | --- | --- | --- | --- | --- | --- |
|  | Overall | | | | | Lagos | | | | | Kano | | | | |
|  | N | % | SE | 95% CI | p-value | N | % | SE | 95% CI | p-value | N | % | SE | 95% CI | p-value |
| Health Center | 206 | 20.4% | 2.2% | (16.4%, 25.0%) |  | 95 | 24.2% | 3.5% | (17.9%, 31.8%) |  | 111 | 17.1% | 2.7% | (12.4%, 23.1%) |  |
|  | | | | | | | | | | | | | | | |
| Drug Shop | 355 | 21.4% | 1.5% | (18.5%, 24.6%) | 0.70 | 171 | 19.3% | 2.2% | (15.3%, 24%) | 0.22 | 184 | 23.4% | 2.1% | (19.4%, 27.9%) | 0.08 |
| Pharmacy | 113 | 8.0% | 1.1% | (6%, 10.4%) | 0.000 | 94 | 7.4% | 1.3% | (5.3%, 10.4%) | 0.000 | 19 | 10.5% | 1.6% | (7.6%, 14.4%) | 0.03 |
| Lab | 100 | 83.0% | 1.5% | (79.8%, 85.8%) | 0.000 | 80 | 85.0% | 1.5% | (81.8%, 87.7%) | 0.000 | 20 | 75.0% | 4.5% | (64.6%, 83.2%) | 0.000 |
| Clinic | 269 | 31.2% | 1.8% | (27.7%, 34.9%) | 0.000 | 203 | 34.7% | 2.4% | (30.1%, 39.5%) | 0.02 | 73 | 21.9% | 2.1% | (18%, 26.3%) | 0.17 |
| CI: 95% confidence interval  SE: Robust standard error | | | | | | | | | | | | | | | |

## Table K: Case 1 – all public DOTS and non-DOTS facility results

| Public Facilities - Case 1: Presumptive Patient Scenario | Public Non DOTS  (All, n=64) | | | Public DOTS  (All, n=142) | | | Public Non DOTS  (Lagos, n=23) | | Public DOTS  (Lagos, n= 72) | | Public Non DOTS  (Kano, n=41) | | Public DOTS (Kano, n=70)) | |
| --- | --- | --- | --- | --- | --- | --- | --- | --- | --- | --- | --- | --- | --- | --- |
|  | % | CI | % | | CI | % | | CI | % | CI | % | CI | % | CI |
| Highest qualification of provider seen |  |  |  | |  |  | |  |  |  |  |  |  |  |
| Consultant | 65.6% | (58.6%, 72%) | 66.2% | | (59.6%, 72.2%) | 43.5% | | (31.2%, 56.6%) | 51.4% | (41.5%, 61.2%) | 78.0% | (69.1%, 85%) | 81.4% | (72.4%, 88%) |
| Nurse | 26.6% | (21%, 33%) | 24.6% | | (19.4%, 30.7%) | 52.2% | | (39.3%, 64.8%) | 40.3% | (31%, 50.4%) | 12.2% | (7.2%, 20%) | 8.6% | (4.4%, 16%) |
| Pharmacist/Drug Shops | 0.0% | (.%, .%) | 0.0% | | (.%, .%) | 0.0% | | (.%, .%) | 0.0% | (.%, .%) | 0.0% | (.%, .%) | 0.0% | (.%, .%) |
| Shop assistant | 0.0% | (.%, .%) | 0.0% | | (.%, .%) | 0.0% | | (.%, .%) | 0.0% | (.%, .%) | 0.0% | (.%, .%) | 0.0% | (.%, .%) |
| Registrar/receptionist | 1.6% | (.5%, 5.1%) | 1.4% | | (.4%, 4.5%) | 0.0% | | (.%, .%) | 1.4% | (.3%, 7.2%) | 2.4% | (.7%, 8%) | 1.4% | (.3%, 7.2%) |
| Laboratory technician | 0.0% | (.%, .%) | 0.0% | | (.%, .%) | 0.0% | | (.%, .%) | 0.0% | (.%, .%) | 0.0% | (.%, .%) | 0.0% | (.%, .%) |
| Other | 6.3% | (3.4%, 11.1%) | 1.4% | | (.4%, 4.4%) | 4.3% | | (1.2%, 14.1%) | 0.0% | (.%, .%) | 7.3% | (3.6%, 14.1%) | 2.9% | (.9%, 8.8%) |
| Don't know | 0.0% | (.%, .%) | 6.3% | | (3.7%, 10.7%) | 0.0% | | (.%, .%) | 6.9% | (3.3%, 14.1%) | 0.0% | (.%, .%) | 5.7% | (2.5%, 12.5%) |
| Gender of provider (highest cadre seen) |  |  |  | |  |  | |  |  |  |  |  |  |  |
| Female | 43.8% | (37.4%, 50.3%) | 52.1% | | (45.9%, 58.3%) | 78.3% | | (65.6%, 87.1%) | 75.0% | (65.4%, 82.6%) | 24.4% | (17.1%, 33.5%) | 28.6% | (20.5%, 38.3%) |
| Male | 56.3% | (49.7%, 62.6%) | 47.9% | | (41.7%, 54.1%) | 21.7% | | (12.9%, 34.4%) | 25.0% | (17.4%, 34.6%) | 75.6% | (66.5%, 82.9%) | 71.4% | (61.7%, 79.5%) |
| Provider confirmed duration of productive cough and one more symptom (Clinical) | 35.9% | (29.1%, 43.5%) | 37.3% | | (30.9%, 44.2%) | 30.4% | | (19.9%, 43.6%) | 45.8% | (36.2%, 55.8%) | 39.0% | (30.2%, 48.7%) | 28.6% | (20.5%, 38.3%) |
| Provider took sputum sample, rec'd chest X-ray, or referred SP for sputum test | 46.9% | (39.5%, 54.4%) | 51.4% | | (44.4%, 58.3%) | 56.5% | | (43.4%, 68.8%) | 52.8% | (42.8%, 62.5%) | 41.5% | (32.4%, 51.1%) | 50.0% | (40.2%, 59.8%) |
| No unnecessary sale of antibiotics, anti-TB drugs, or steroids | 67.2% | (60%, 73.7%) | 69.7% | | (63.1%, 75.6%) | 82.6% | | (70.5%, 90.4%) | 79.2% | (69.9%, 86.2%) | 58.5% | (48.9%, 67.6%) | 60.0% | (50%, 69.2%) |
| Correct Management (met all 3 criteria) | 21.9% | (16.3%, 28.7%) | 19.7% | | (14.7%, 25.9%) | 26.1% | | (16.3%, 39%) | 23.6% | (16.2%, 33.1%) | 19.5% | (13%, 28.2%) | 15.7% | (9.7%, 24.4%) |
| Met one of the three criteria | 43.8% | (36.4%, 51.3%) | 33.1% | | (26.9%, 40%) | 39.1% | | (27.3%, 52.3%) | 34.7% | (25.9%, 44.7%) | 46.3% | (37%, 55.9%) | 31.4% | (23%, 41.3%) |
| Provider confirmed duration of productive cough and one more symptom (Clinical) | 14.3% | (8%, 24.1%) | 10.6% | | (5.1%, 20.8%) | 0.0% | | (.%, .%) | 12.0% | (4.6%, 27.9%) | 21.1% | (11.8%, 34.7%) | 9.1% | (2.8%, 25.6%) |
| Provider took sputum sample, rec'd chest X-ray, or referred SP for sputum test | 14.3% | (8%, 24.3%) | 17.0% | | (9.9%, 27.8%) | 11.1% | | (3.2%, 32.1%) | 4.0% | (0.7%, 19.1%) | 15.8% | (8%, 28.9%) | 31.8% | (17.9%, 50%) |
| No unnecessary sale of antibiotics, anti-TB drugs, or steroids | 71.4% | (60.3%, 80.5%) | 72.3% | | (60.5%, 81.7%) | 88.9% | | (67.9%, 96.8%) | 84.0% | (67.5%, 93%) | 63.2% | (48.9%, 75.4%) | 59.1% | (41.2%, 74.8%) |
| Met two of the three criteria | 20.3% | (14.9%, 27%) | 33.1% | | (26.9%, 40%) | 26.1% | | (16.3%, 39%) | 36.1% | (27.1%, 46.2%) | 17.1% | (11%, 25.5%) | 30.0% | (21.7%, 39.8%) |
| Duration/productive cough and sputum sample | 30.8% | (17.8%, 47.8%) | 21.3% | | (13%, 32.9%) | 16.7% | | (4.8%, 44.1%) | 26.9% | (14.8%, 43.8%) | 42.9% | (22.7%, 65.7%) | 14.3% | (5.6%, 32%) |
| Sputum sample and no unnecessary sale | 61.5% | (44.8%, 75.9%) | 57.4% | | (45.3%, 68.8%) | 83.3% | | (55.9%, 95.2%) | 50.0% | (33.9%, 66.1%) | 42.9% | (22.7%, 65.7%) | 66.7% | (48.1%, 81.2%) |
| Duration/productive cough and no unnecessary sale | 7.7% | (2.3%, 22.6%) | 21.3% | | (13%, 32.9%) | 0.0% | | (.%, .%) | 23.1% | (12%, 39.8%) | 14.3% | (4.2%, 38.5%) | 19.0% | (8.5%, 37.2%) |
| Met no criteria | 14.00% | (9.6%, 20.1%) | 14.10% | | (9.4%, 18.6%) | 8.70% | | (3.6%, 19.4%) | 5.60% | (1.6%, 10.6%) | 17.10% | (11.0%, 25.5%) | 22.90% | (15.6%, 32.2%) |
| Met at least 1 criteria | 86.0% | (79.9%, 90.4%) | 85.9% | | (81.4%, 90.6%) | 91.3% | | (80.6%, 96.4%) | 94.4% | (89.4%, 98.4%) | 82.9% | (74.5%, 89.0%) | 77.1% | (67.8%, 84.4%) |
| CI: 95% confidence interval | | | | | | | | | | | | | | |

## Table L: Case 1 Correlation Tables, Public DOTS and Non-DOTS Clinical Facilities

|  | Public Non DOTS (All, n=64) | | | |
| --- | --- | --- | --- | --- |
|  | Duration of cough | Sputum sample, chest X-ray, or referred SP for sputum test | No unnecessary sale of antibiotics, anti-TB drugs, or steroids | Successful completion of scenario |
| Duration of cough | 1.00 |  |  |  |
| Sputum sample, chest X-ray, or referred SP for sputum test | 0.69 | 1.00 |  |  |
| No unnecessary sale of antibiotics, anti-TB drugs, or steroids | -0.05 | 0.20 | 1.00 |  |
| Successful completion of scenario | 1.00 | 1.00 | 1.00 | 1.00 |
|  |  |  |  |  |
|  | Public DOTS (All, n=142) | | | |
|  | Duration of cough | Sputum sample, chest X-ray, or referred SP for sputum test | No unnecessary sale of antibiotics, anti-TB drugs, or steroids | Successful completion of scenario |
| Duration of cough | 1.00 |  |  |  |
| Sputum sample, chest X-ray, or referred SP for sputum test | 0.48 | 1.00 |  |  |
| No unnecessary sale of antibiotics, anti-TB drugs, or steroids | 0.06 | 0.21 | 1.00 |  |
| Successful completion of scenario | 1.00 | 1.00 | 1.00 | 1.00 |
