# Supplementary material for: Tuberculosis care quality in urban Nigeria: A cross-sectional study of adherence to screening and treatment initiation guidelines in multi-cadre networks of private health service providers
Source: PLOS Glob Public Health. 2022 Jan 6;2(1):e0000150. doi: 10.1371/journal.pgph.0000150 (PMC10021846; doi:10.1371/journal.pgph.0000150)
Supplement: S4 Annex — (DOCX) [file pgph.0000150.s004.docx]

# **S4 Annex: Case 2 detailed results tables, including state-specific results**

The tables below provide detailed results for Case 2 (the confirmed TB SP scenario). Table A presents for all private (SHOPS Plus) facilities (overall and for each state) that were visited by SPs for this study. Table B shows results for all public DOTS facilities (overall and for each state). Table C shows results for a correlation analysis of the component elements. Table D shows Case 2 regression results. Table E shows Case 2 results for all private SHOPS Plus facilities benchmarked to all public DOTS facilities. Table F shows results from a sensitivity analysis we conducted where the criterion related to prescriptions was narrowed so that it would only apply to prescriptions of steroids and fluroquinolones (and not other broad- and narrow-spectrum antibiotics).

## Table A: Case 2 - Confirmed Patient Scenario Results, SHOPS Plus Clinical Facilities

| SHOPS Plus Clinical Case 2: Confirmed Patient Scenario | SHOPS Plus Clinical (All, n=228) | | SHOPS Plus Clinical (Lagos, n=174) | | SHOPS Plus Clinical (Kano, n=54) | |
| --- | --- | --- | --- | --- | --- | --- |
|  | % | CI | % | CI | % | CI |
| Highest qualification of provider seen |  |  |  |  |  |  |
| Consultant | 87.5% | (84.4%, 90%) | 87.4% | (83.5%, 90.4%) | 87.7% | (82.7%, 91.4%) |
| Nurse | 10.2% | (7.9%, 13.1%) | 11.1% | (8.2%, 14.7%) | 7.7% | (4.8%, 12%) |
| Pharmacist/PPMVs | 0.4% | (.1%, 1.1%) | 0.0% | (.%, .%) | 1.5% | (.5%, 4.4%) |
| Shop assistant | 0.4% | (.1%, 1.1%) | 0.0% | (.%, .%) | 1.5% | (.5%, 4.4%) |
| Registrar/receptionist | 0.8% | (.3%, 2.1%) | 1.1% | (.4%, 2.8%) | 0.0% | (.%, .%) |
| Laboratory technician | 0.0% | (.%, .%) | 0.0% | (.%, .%) | 0.0% | (.%, .%) |
| Other | 0.4% | (.1%, 1.1%) | 0.0% | (.%, .%) | 1.5% | (.5%, 4.4%) |
| Don't know | 0.4% | (.1%, 1.6%) | 0.5% | (.1%, 2.1%) | 0.0% | (.%, .%) |
| Gender of provider (highest cadre seen) |  |  |  |  |  |  |
| Female | 28.6% | (25%, 32.6%) | 30.5% | (26%, 35.5%) | 23.1% | (18%, 29.1%) |
| Male | 71.4% | (67.4%, 75%) | 69.5% | (64.5%, 74%) | 76.9% | (70.9%, 82%) |
| Confirmation of diagnosis | 72.6% | (68.2%, 76.7%) | 70.7% | (65.1%, 75.8%) | 78.0% | (70.9%, 83.7%) |
| Explanation of disease and treatment | 44.8% | (40.2%, 49.6%) | 49.4% | (43.5%, 55.3%) | 32.2% | (25.5%, 39.8%) |
| Requests patient to identify a treatment supporter | 37.2% | (32.7%, 41.9%) | 39.0% | (33.5%, 44.9%) | 32.2% | (25.5%, 39.8%) |
| No prescription of non-TB antibiotics, steroids | 83.4% | (79.5%, 86.7%) | 82.9% | (78%, 86.9%) | 84.7% | (78.4%, 89.5%) |
| Prescription/provision of TB drugs for treatment/treatment initiation OR request to come back with the treatment supporter | 70.0% | (65.7%, 73.9%) | 78.7% | (73.4%, 83.1%) | 45.8% | (38.2%, 53.5%) |
| Correct Management (met all 5 criteria) | 18.4% | (15%, 22.4%) | 19.5% | (15.3%, 24.6%) | 15.3% | (10.5%, 21.6%) |
| Met one of the five criteria | 11.4% | (9%, 14.2%) | 9.5% | (6.9%, 12.9%) | 16.9% | (12.5%, 22.5%) |
| #1. Confirmation of diagnosis | 0.0% | (.%, .%) | 0.0% | (.%, .%) | 0.0% | (.%, .%) |
| #2. Explanation of disease and treatment | 0.0% | (.%, .%) | 0.0% | (.%, .%) | 0.0% | (.%, .%) |
| #3. Requests SP to identify a treatment supporter | 0.0% | (.%, .%) | 0.0% | (.%, .%) | 0.0% | (.%, .%) |
| #4. No prescription of non-TB antibiotics, steroids | 86.2% | (75.4%, 92.7%) | 83.3% | (67.1%, 92.5%) | 90.9% | (76.6%, 96.8%) |
| #5. Prescription/provision of TB drugs for treatment/treatment initiation OR request to come back with the treatment supporter | 13.8% | (7.3%, 24.6%) | 16.7% | (7.5%, 32.9%) | 9.1% | (3.2%, 23.4%) |
| Met two of the five criteria | 19.2% | (16.2%, 22.6%) | 16.8% | (13.3%, 21.1%) | 26.2% | (20.8%, 32.3%) |
| Most common two criteria met |  | #1; #4 |  | #4, #5 |  | #1; #4 |
| Met three of the five criteria | 19.2% | (16.1%, 22.7%) | 18.9% | (15.2%, 23.3%) | 20.0% | (15.2%, 25.8%) |
| Most common three criteria met |  | #1; #4; #5 |  | #1; #4; #5 |  | #1; #4; #5 |
| Met four of the five criteria | 20.4% | (17.2%, 24%) | 23.2% | (19.1%, 27.8%) | 12.3% | (8.6%, 17.3%) |
|  | #1; #2; #4; #5 | | #1; #2; #4; #5 | | #1; #2; #3; #5 | |
| CI: 95% confidence interval | | | | | | |

## Table B: Case 2 - Confirmed Patient Scenario Results, Public DOTS Facilities

| SHOPS Plus Clinical and Public DOTS - Case 2: Confirmed Patient Scenario | Public DOTS  (All, n=119) | | Public DOTS  (Lagos, n=60) | | Public DOTS  (Kano, n=59) | |
| --- | --- | --- | --- | --- | --- | --- |
|  | % | CI | % | CI | % | CI |
| Highest qualification of provider seen |  |  |  |  |  |  |
| Consultant | 52.7% | (45.3%, 60%) | 43.8% | (33.4%, 54.7%) | 61.5% | (50.8%, 71.2%) |
| Nurse | 37.2% | (30.4%, 44.5%) | 48.4% | (37.8%, 59.2%) | 26.2% | (17.9%, 36.5%) |
| Pharmacist/PPMVs | 0.8% | (.1%, 4.1%) | 0.0% | (.%, .%) | 1.5% | (.3%, 8.1%) |
| Shop assistant | 0.0% | (.%, .%) | 0.0% | (.%, .%) | 0.0% | (.%, .%) |
| Registrar/receptionist | 0.8% | (.1%, 4.2%) | 1.6% | (.3%, 8.4%) | 0.0% | (.%, .%) |
| Laboratory technician | 2.3% | (.9%, 6%) | 0.0% | (.%, .%) | 4.6% | (1.7%, 11.8%) |
| Other | 1.6% | (.5%, 5.1%) | 3.1% | (.9%, 10.1%) | 0.0% | (.%, .%) |
| Don't know | 4.7% | (2.3%, 9%) | 3.1% | (.9%, 10.1%) | 6.2% | (2.6%, 13.7%) |
| Gender of provider (highest cadre seen) |  |  |  |  |  |  |
| Female | 50.4% | (44%, 56.8%) | 76.6% | (66.2%, 84.5%) | 24.6% | (16.6%, 34.9%) |
| Male | 49.6% | (43.2%, 56%) | 23.4% | (15.5%, 33.8%) | 75.4% | (65.1%, 83.4%) |
| Confirmation of diagnosis | 66.4% | (58.5%, 73.4%) | 68.4% | (56.6%, 78.2%) | 64.5% | (53.4%, 74.2%) |
| Explanation of disease and treatment | 31.1% | (24.3%, 38.8%) | 26.3% | (17.3%, 37.9%) | 35.5% | (25.8%, 46.6%) |
| Requests patient to identify a treatment supporter | 29.4% | (22.7%, 37.1%) | 31.6% | (21.8%, 43.4%) | 27.4% | (18.7%, 38.2%) |
| No prescription of non-TB antibiotics, steroids | 88.2% | (82.1%, 92.5%) | 91.2% | (81.9%, 96%) | 85.5% | (75.9%, 91.7%) |
| Prescription/provision of TB drugs for treatment/treatment initiation OR request to come back the next day with the treatment supporter | 59.7% | (51.7%, 67.2%) | 56.1% | (44.3%, 67.3%) | 62.9% | (51.8%, 72.8%) |
| Correct Management (met all 5 criteria) | 13.4% | (8.9%, 19.8%) | 10.5% | (5.2%, 20.2%) | 16.1% | (9.6%, 25.9%) |
| Met one of the five criteria | 20.2% | (14.8%, 26.8%) | 17.2% | (10.4%, 27%) | 23.1% | (15.3%, 33.2%) |
| #1. Confirmation of diagnosis | 0.0% | (.%, .%) | 0.0% | (.%, .%) | 0.0% | (.%, .%) |
| #2. Explanation of disease and treatment | 0.0% | (.%, .%) | 0.0% | (.%, .%) | 0.0% | (.%, .%) |
| #3. Requests SP to identify a treatment supporter | 0.0% | (.%, .%) | 0.0% | (.%, .%) | 0.0% | (.%, .%) |
| #4. No prescription of non-TB antibiotics, steroids | 88.5% | (72.9%, 95.6%) | 90.9% | (61.7%, 98.4%) | 86.7% | (63.9%, 96%) |
| #5. Prescription/provision of TB drugs for treatment/treatment initiation OR request to come back the next day with the treatment supporter | 11.5% | (4.4%, 27.1%) | 9.1% | (1.6%, 38.3%) | 13.3% | (4%, 36.1%) |
| Met two of the five criteria | 23.3% | (17.5%, 30.2%) | 21.9% | (14.2%, 32.2%) | 24.6% | (16.6%, 34.9%) |
| Most common two criteria met |  | #1; #4 |  | #1; #4 |  | #1; #4 |
| Met three of the five criteria | 14.7% | (10.2%, 20.8%) | 20.3% | (12.9%, 30.4%) | 9.2% | (4.6%, 17.5%) |
| Most common three criteria met |  | #1; #4; #5 |  | #1; #4; #5 |  | #1; #4; #5 |
| Met four of the five criteria | 20.2% | (14.8%, 26.9%) | 18.8% | (11.7%, 28.7%) | 21.5% | (14.1%, 31.5%) |
|  | #1; #2; #4; #5 | | (#1; #3; #4; #5) AND (#2; #3; #4; #5) | | #1; #2; #3; #5 | |
| CI: 95% confidence interval | | | | | | |

## Table C: Case 2 Correlation Tables, Private and Public DOTS Facilities

| SHOPS Plus Clinical (n=228) | | | | | | |
| --- | --- | --- | --- | --- | --- | --- |
|  | Confirmation of diagnosis | Explanation of disease and treatment | Requests patient to identify a treatment supporter | No prescription of non-TB antibiotics, steroids | Prescription/provision of TB drugs for treatment/treatment initiation OR request to come back the next day with the treatment supporter | Provider successfully treated SP |
| Confirmation of diagnosis | 1.00 |  |  |  |  |  |
| Explanation of disease and treatment | 0.65 | 1.00 |  |  |  |  |
| Requests patient to identify a treatment supporter | 0.75 | 0.83 | 1.00 |  |  |  |
| No prescription of non-TB antibiotics, steroids | -0.06 | -0.37 | -0.53 | 1.00 |  |  |
| Prescription/provision of TB drugs for treatment/treatment initiation OR request to come back the next day with the treatment supporter | 0.38 | 0.55 | 0.52 | -0.23 | 1.00 |  |
| Provider successfully treated SP | 1.00 | 1.00 | 1.00 | 1.00 | 1.00 | 1.00 |
| Public DOTS (n=119) | | | | | | |
|  | Confirmation of diagnosis | Explanation of disease and treatment | Requests patient to identify a treatment supporter | No prescription of non-TB antibiotics, steroids | Prescription/provision of TB drugs for treatment/treatment initiation OR request to come back the next day with the treatment supporter | Provider successfully treated SP |
| Confirmation of diagnosis | 1.00 |  |  |  |  |  |
| Explanation of disease and treatment | 0.80 | 1.00 |  |  |  |  |
| Requests patient to identify a treatment supporter | 0.79 | 0.75 | 1.00 |  |  |  |
| No prescription of non-TB antibiotics, steroids | 0.03 | -0.19 | -0.32 | 1.00 |  |  |
| Prescription/provision of TB drugs for treatment/treatment initiation OR request to come back the next day with the treatment supporter | 0.50 | 0.67 | 0.71 | -0.44 | 1.00 |  |
| Provider successfully treated SP | 1.00 | 1.00 | 1.00 | 1.00 | 1.00 | 1.00 |

## Table D: Case 2 Regression Results

| **Variable** | **Steps 1-5 of Case 2 Correct Management Sequence** | | | | | | | | | | | | | | | | | | | | | | | | | | | | | | | | | | | | | | | | | | | | | |  | | | | | | | | | |
| --- | --- | --- | --- | --- | --- | --- | --- | --- | --- | --- | --- | --- | --- | --- | --- | --- | --- | --- | --- | --- | --- | --- | --- | --- | --- | --- | --- | --- | --- | --- | --- | --- | --- | --- | --- | --- | --- | --- | --- | --- | --- | --- | --- | --- | --- | --- | --- | --- | --- | --- | --- | --- | --- | --- | --- | --- |
|  | Confirmation of diagnosis | | | | | | Explanation of disease and treatment | | | | | | | | | | Requests patient to identify a treatment supporter | | | | | | | | | | No prescription of non-TB antibiotics, steroids | | | | | | | | | | Prescription/provision of TB drugs for treatment/ treatment initiation OR request to come back the next day with the treatment supporter | | | | | | | | | | Provider successfully treated mystery client | | | | | | | | | |
|  | OR | SE | 95% CI | | | | OR | SE | | | 95% CI | | | | | | OR | SE | | | 95% CI | | | | | | OR | SE | | | 95% CI | | | | | | OR | SE | | | 95% CI | | | | | | OR | SE | | | 95% CI | | | | | |
|  |  |  | Lower | | Upper | |  |  |  |  | Lower | | | Upper | | |  |  |  |  | Lower | | | Upper | | |  |  |  |  | Lower | | | Upper | | |  |  |  |  | Lower | | | Upper | | |  |  |  |  | r | | Lower Upper | | | |
| State |  |  | |  | |  |  | |  | | |  | | |  | |  | |  | | |  | | |  | |  | |  | | |  | | |  | |  | |  | | |  | | |  | |  | |  | | |  | | |  | |
| Lagos | 0.76 | 0.51 | | 0.2 | | 2.84 | 1.69 | | | 0.57 | | | 0.87 | | | 3.27 | 1.19 | | | 0.4 | | | 0.62 | | | 2.3 | 0.91 | | | 0.39 | | | 0.39 | | | 2.12 | 4.49 | | | 1.95*** | | | 1.92 | | | 10.54 | 1.26 | | | 0.57 | | | | 0.52 | | 3.05 |
| Kano (reference) |  | . | |  | |  |  | | | . | | |  | | |  |  | | | . | | |  | | |  |  | | | . | | |  | | |  |  | | | . | | |  | | |  |  | | | . | | | |  | |  |
| Gender: Provider/client |  | . | |  | |  |  | | | . | | |  | | |  |  | | | . | | |  | | |  |  | | | . | | |  | | |  |  | | | . | | |  | | |  |  | | | . | | | |  | |  |
| Female/Female | 0.73 | 0.67 | | 0.12 | | 4.44 | 3.21 | | | 1.49* | | | 1.29 | | | 7.97 | 1.54 | | | 0.67 | | | 0.65 | | | 3.62 | 0.87 | | | 0.48 | | | 0.3 | | | 2.57 | 1.57 | | | 0.94 | | | 0.48 | | | 5.1 | 1.43 | | | 0.64 | | | | 0.6 | | 3.44 |
| Male/Female | 1.38 | 1.24 | | 0.23 | | 8.08 | 1.68 | | | 0.56 | | | 0.88 | | | 3.23 | 1.19 | | | 0.39 | | | 0.63 | | | 2.28 | 1.12 | | | 0.47 | | | 0.49 | | | 2.53 | 1.15 | | | 0.52 | | | 0.47 | | | 2.8 |  | | | . | | | |  | |  |
| Female/Male | 0.86 | 0.63 | | 0.2 | | 3.64 | 0.9 | | | 0.56 | | | 0.27 | | | 3.05 | 0.56 | | | 0.37 | | | 0.16 | | | 2.01 | 3.45 | | | 3.81 | | | 0.4 | | | 30.06 | 1.06 | | | 0.7 | | | 0.29 | | | 3.84 |  | | | . | | | |  | |  |
| Male/Male (reference) |  | . | |  | |  |  | | | . | | |  | | |  |  | | | . | | |  | | |  |  | | | . | | |  | | |  |  | | | . | | |  | | |  | 1.28 | | | 0.51 | | | | 0.59 | | 2.79 |
| Highest position of provider(s) seen |  | . | |  | |  |  | | | . | | |  | | |  |  | | | . | | |  | | |  |  | | | . | | |  | | |  |  | | | . | | |  | | |  |  | | | . | | | |  | |  |
| Consultant | 2.71 | 1.72 | | 0.78 | | 9.42 | 3.42 | | | 1.92* | | | 1.14 | | | 10.29 | 1.82 | | | 0.99 | | | 0.62 | | | 5.3 | 0.44 | | | 0.37 | | | 0.08 | | | 2.32 | 1.68 | | | 0.98 | | | 0.54 | | | 5.25 |  | | | . | | | |  | |  |
| Other than consultant (reference) |  | . | |  | |  |  | | | . | | |  | | |  |  | | | . | | |  | | |  |  | | | . | | |  | | |  |  | | | . | | |  | | |  | 1.04 | | | 0.63 | | | | 0.32 | | 3.4 |
| Number of patients in waiting room (average at start and end of visit) |  | . | |  | |  |  | | | . | | |  | | |  |  | | | . | | |  | | |  |  | | | . | | |  | | |  |  | | | . | | |  | | |  |  | | | . | | | |  | |  |
| Less than 1 (reference) |  | . | |  | |  |  | | | . | | |  | | |  |  | | | . | | |  | | |  |  | | | . | | |  | | |  |  | | | . | | |  | | |  |  | | | . | | | |  | |  |
| 1 to <2 | 1.69 | 0.86 | | 0.62 | | 4.57 | 1.12 | | | 0.42 | | | 0.53 | | | 2.35 | 1.29 | | | 0.48 | | | 0.62 | | | 2.68 | 1.56 | | | 0.8 | | | 0.57 | | | 4.29 | 1.04 | | | 0.45 | | | 0.45 | | | 2.41 | 1.19 | | | 0.54 | | | | 0.49 | | 2.92 |
| 2 to <6 | 1.64 | 0.76 | | 0.66 | | 4.04 | 1.87 | | | 0.66 | | | 0.93 | | | 3.74 | 1.18 | | | 0.41 | | | 0.59 | | | 2.34 | 1.11 | | | 0.49 | | | 0.47 | | | 2.61 | 2.02 | | | 0.88 | | | 0.87 | | | 4.74 | 0.99 | | | 0.44 | | | | 0.41 | | 2.36 |
| 6+ | 1.01 | 0.67 | | 0.27 | | 3.7 | 0.8 | | | 0.43 | | | 0.28 | | | 2.28 | 0.81 | | | 0.44 | | | 0.28 | | | 2.36 | 2.01 | | | 1.6 | | | 0.42 | | | 9.58 | 0.63 | | | 0.35 | | | 0.21 | | | 1.88 | 0.48 | | | 0.39 | | | | 0.1 | | 2.36 |
| OR: Adjusted odds ratio; SE: Robust Standard Error; CI: Confidence Interval;  * p<0.05; ** p<0.01; *** p<0.001 | | | | | | | | | | | | | | | | | | | | | | | | | | | | | | | | | | | | | | | | | | | | | | | | | | | | | | | | |

## Table E: Benchmarking Private Providers’ SP Management - Case 2 Confirmed Patients

| Facility Network | Provider type | Provider successfully treated mystery client | | | | | | | | | | | | | | |
| --- | --- | --- | --- | --- | --- | --- | --- | --- | --- | --- | --- | --- | --- | --- | --- | --- |
|  |  | Overall | | | | | Lagos | | | | | Kano | | | | |
|  |  | N | % | SE | 95% CI | p-value | N | % | SE | 95% CI | p-value | N | % | SE | 95% CI | p-value |
| Public DOTS | Health Center | 119 | 13.4% | 2.7% | (8.9%, 19.8%) |  | 60 | 10.5% | 3.6% | (5.2%, 20.2%) |  | 59 | 16.1% | 4.0% | (9.6%, 25.9%) |  |
|  | | | | | | | | | | | | | | | | |
| SHOPS Plus | Clinic | 228 | 18.4% | 1.9% | (15%, 22.4%) | 0.16 | 174 | 19.5% | 2.4% | (15.3%, 24.6%) | 0.07 | 54 | 15.3% | 2.8% | (10.5%, 21.6%) | 0.86 |
| CI: 95% confidence interval  SE: Robust Standard Error | | | | | | | | | | | | | | | | |

## Table F: Sensitivity Analysis w/ relaxed Rx criterion- Case 2 Confirmed Patients

| SHOPS Plus Clinical and Public DOTS - Confirmed Patient Scenario | SHOPS Plus Clinical (All, n=228) | | SHOPS Plus Clinical (Lagos, n=174) | | SHOPS Plus Clinical (Kano, n=54) | | Public DOTS (All, n=129) | | Public DOTS (Lagos, n=60) | | Public DOTS (Kano, n=59) | |
| --- | --- | --- | --- | --- | --- | --- | --- | --- | --- | --- | --- | --- |
|  | % | CI | % | CI | % | CI | % | CI | % | CI | % | CI |
| Confirmation of diagnosis | 72.6% | (68.2%, 76.7%) | 70.7% | (65.1%, 75.8%) | 78.0% | (70.9%, 83.7%) | 66.4% | (58.5%, 73.4%) | 68.4% | (56.6%, 78.2%) | 64.5% | (53.4%, 74.2%) |
| Explanation of disease and treatment | 44.8% | (40.2%, 49.6%) | 49.4% | (43.5%, 55.3%) | 32.2% | (25.5%, 39.8%) | 31.1% | (24.3%, 38.8%) | 26.3% | (17.3%, 37.9%) | 35.5% | (25.8%, 46.6%) |
| Requests patient to identify a treatment supporter | 37.2% | (32.7%, 41.9%) | 39.0% | (33.5%, 44.9%) | 32.2% | (25.5%, 39.8%) | 29.4% | (22.7%, 37.1%) | 31.6% | (21.8%, 43.4%) | 27.4% | (18.7%, 38.2%) |
| No prescription of fluroquinolones or steroids | 91.5% | (88.4%, 93.8%) | 90.9% | (86.8%, 93.7%) | 93.2% | (88.2%, 96.2%) | 95.8% | (91.2%, 98.1%) | 94.7% | (86.2%, 98.1%) | 96.8% | (89.6%, 99%) |
| Prescription/provision of TB drugs for treatment/treatment initiation OR request to come back the next day with the treatment supporter | 70.0% | (65.7%, 73.9%) | 78.7% | (73.4%, 83.1%) | 45.8% | (38.2%, 53.5%) | 59.7% | (51.7%, 67.2%) | 56.1% | (44.3%, 67.3%) | 62.9% | (51.8%, 72.8%) |
| Correct SP management | 22.4% | (18.7%, 26.7%) | 23.8% | (19.1%, 29.2%) | 18.6% | (13.4%, 25.4%) | 16.8% | (11.7%, 23.5%) | 10.5% | (5.2%, 20.2%) | 22.6% | (14.7%, 33%) |
