# Supplementary material for: Tuberculosis care quality in urban Nigeria: A cross-sectional study of adherence to screening and treatment initiation guidelines in multi-cadre networks of private health service providers
Source: PLOS Glob Public Health. 2022 Jan 6;2(1):e0000150. doi: 10.1371/journal.pgph.0000150 (PMC10021846; doi:10.1371/journal.pgph.0000150)
Supplement: S5 Annex — (DOCX) [file pgph.0000150.s005.docx]

# **Annex S5: Comparing results of TB quality of care studies that use SP survey designs**

Most TB-related SP studies have examined case management in clinical facilities and at a minimum, explored quality using a “textbook” presumptive TB SP scenario. For all the studies listed in the table below, the SP presented in the same way as SPs did for Case 1, with one exception. In Christian et al.’s 2018 South Africa study in public clinical facilities, SPs were instructed to tell the provider “I think I may have TB” if the provider did not ask questions after the SP’s initial presentation of a chronic cough and fever—this may partially explain why successful outcomes in this study are higher than in other studies. All other cases were like ours (i.e., SPs were only allowed to answer questions asked directly to them after giving their opening statement).

This table below shows results for other SP studies lenient definition of correct management, which corresponds to one of the criterion we used to calculate correct management in this study. As the table shows, the results from this study (Rosapep et al, 2022) are neither the lowest nor the highest of the other TB SP studies implemented in clinical facilities to date, though when compared to other private sector providers, more Nigerian providers took or referred for a sputum sample or chest x-ray.

| Study | Country | Sector | Lenient Definition of Correct Management | Appropriate TB Diagnostic or Referral  (% of SPs) |
| --- | --- | --- | --- | --- |
| Christian et al., 2018 | Urban South Africa | Public Clinical | Should be asked to undergo a sputum and HIV test | 84% |
| Daniels et al., 2017 | Urban Kenya | Public and Private Clinical | Should be asked to undergo a sputum test | 79% public  33% private |
| Rosapep et al., 2022 | Urban Nigeria | Public and Private Clinical | Referral, chest x-ray, and/or sputum test | 56% private (Lagos 58%, Kano 49%)  51% public (Lagos 53%, Kano 50%) |
| Boffa et al., 2021 | Urban South Africa | Private Clinical | Referral, chest x-ray, and/or sputum test | 43% |
| Sylvia et al., 2017 | Rural China | Public Clinical | Referral, chest x-ray, and/or sputum test | 41% |
| Kwan, et al., 2018 | Urban India | Private Clinical | Referral, chest x-ray, and/or sputum test | 35% |
| Das et al., 2015 | Urban India | Private Clinical | Referral, chest x-ray, and/or sputum test | 16% |
