# Supplementary material for: Tuberculosis care quality in urban Nigeria: A cross-sectional study of adherence to screening and treatment initiation guidelines in multi-cadre networks of private health service providers
Source: PLOS Glob Public Health. 2022 Jan 6;2(1):e0000150. doi: 10.1371/journal.pgph.0000150 (PMC10021846; doi:10.1371/journal.pgph.0000150)
Supplement: Attachment — Submitted filename: 4. Response to Reviewers_Nigeria TB SP.docx [file pgph.0000150.s006.docx]

**Responses to Reviewers**

**Journal Requirements:**

1. *Please update the completed 'Competing Interests' statement, including any COIs declared by your co-authors. If you have no competing interests to declare, please state "The authors have declared that no competing interests exist". Otherwise please declare all competing interests beginning with the statement "I have read the journal's policy and the authors of this manuscript have the following competing interests:"*

a. During our initial submission we had already indicated some of the authors do have competing interest. In the resubmission we have included the same statement but have added the following statement: *“I have read the journal’s policy and the authors of this manuscript have the following competing interests”.*

2. *In the online submission form, you indicated that "All data relevant to the study are freely available to those who register as users of USAID’s Development Data Library, which can be found at:* *<https://data.usaid.gov/Tuberculosis/Nigeria-Tuberculosis-Program-Quality-of-Care-Study/f8bn-qej9>".All PLOS journals now require all data underlying the findings described in their manuscript to be freely available to other researchers, either 1. In a public repository, 2. Within the manuscript itself, or 3. Uploaded as supplementary information. This policy applies to all data except where public deposition would breach compliance with the protocol approved by your research ethics board. If your data cannot be made publicly available for ethical or legal reasons (e.g., public availability would compromise patient privacy), please explain your reasons by return email and your exemption request will be escalated to the editor for approval. Your exemption request will be handled independently and will not hold up the peer review process, but will need to be resolved should your manuscript be accepted for publication. One of the Editorial team will then be in touch if there are any issues.*
   a. We believe that we have already met this requirement. The dataset that we generated and analyzed for this article is already publicly available on the USAID Development Data Library (DDL) at the link we have previously provided. The DDL is a public repository maintained by USAID.  Anyone can access this data at no cost once they have registered for a free user account on the DDL.  We will emphasize this point in our re-submission to make it clear that registering as a user for the DDL is free and therefore the data are freely available to other researchers.

**Reviewer #1**

1. *I'm not yet convinced of the value added by the bottleneck analysis. My concern is that the events of the ideal diagnostic cascade as outline by the authors in a monotonic sequential way (ie if you get one wrong you get all the remaining steps wrong). The bottleneck analysis disregards any provide action after their first failed step. This given the impression of very poor providers performance even though that the authors point out that for case 2 70% of providers do the most important things: initiate TB treatment. If a provider doesn't ask more questions about symptoms but does recommend a diagnostic and avoids unnecessary drugs, they're virtually invisible in the bottleneck figures.*

Thank you for this observation, it has helped us see that more explanation is necessary to properly interpret the bottleneck analysis. We have added more explanation in the manuscript at Line 309-331. Based on the current NTBLCP guidelines for presumptive and confirmed patient management, there is an ordered sequence of actions that providers are recommended to follow, and our bottleneck analysis is informed by this sequence. The overall case management and individual case element results, which are presented at the beginning of each case result section present results which are agnostic to any sequence.

2. *Additionally, I am confused about why you have selected certain drop offs to be labelled as bottlenecks over others. For example in Figure 2, The 27% failure at step 1 is labelled a bottleneck but the 30% drop off at step 4 is not. What is the criteria for bottleneck?*

Thank you for pointing out this error in our figure. We have corrected that error in Figure 2 and added more explanatory language for how we defined bottlenecks. See line 320-324.

3. *Would a correlation analysis not provide similar data that is agnostic to the sequence of steps? As an example of what I'm finding confusing, in line 417 the authors state "our bottleneck analysis shows that the majority of PPMVs and CPs who adhere to the testing protocol also adhere to the final stage of correct management by not dispensing unnecessary medications" But is is the rate of inappropriate dispensing difference between those who do and do not adhere to testing protocol? ie is there actually an impact of step 1 on step 3? In short, I am not clear on what additional useful, actionable information is offered by this analysis. Currently, it muddies the water for me in light of the authors other comments that critical actions (referring for dx, or initiating trt) are relatively common.*

We respectfully disagree that the bottleneck analysis does not offer additional insights into the initial management of presumptive and confirmed TB patients, especially as there is an implied sequence of case management of elements as outlined in the NTPLCP guidelines. The bottleneck analysis is designed to be utilization focused, and in our experience, it was a useful way to present the data for program stakeholders in Nigeria to be able to more-easily pinpoint actions that a disproportionate number of providers had trouble demonstrating in interactions with SPs. This facilitated further conversation on where further remediation action for the program.

As we’ve stated above, we’ve added additional explanation in the manuscript to better describe our bottleneck approach, but we’ve also added an additional correlation analysis as suggested. We’ve included these in the Annexes (See Annex S3 Table H and Annex S4 Table C ) and described the results in the manuscript (see Lines 422-432, and 490-497). In general, the correlations show that providers that successfully completed one case element tend to complete other elements. The exception is the Case 2 element of not prescribing non-TB antibiotics or steroids to treatment initiation patients, as the correlations indicate that clinical providers who successfully completed other elements of Case 2 were in general more likely to prescribe inappropriate drugs. However, because the number and proportion of providers prescribing inappropriate drugs is small it is hard to meaningfully interpret this correlation.

*4. Line 105, Please provide rationale for choosing Kano and Lagos in addition to the description you have provided of the setting*

a. Lagos and Kano were purposively selected as these were the only two states in which SHOPS Plus was implementing its network model to support private sector provision of TB screening, diagnostic, and treatment services (See Line 129). Since our study primarily focuses on the quality of care of TB services provided by trained providers in SHOPS Plus networks there were no other states in which this study could have been implemented at the time. We’ve included more information on the program background in the manuscript.

*5. line 146, the levels of the substrata are given in Table 2 but it would also be helpful to list and define them here*

a. Thank you for making this suggestion. We have added detail on the substrata in the body of the revised manuscript. See Line 212-218.

*6. Line 149, what specific estimates were the sample size calculations powered for? I believe this was the MOE around the proportion of correct management?*

a. We ran sample size calculations for each of the substrata. We used a 95% level of confidence (α = 0.05) for all calculations, and set the proportion of providers following appropriate patient management protocols at p=0.5 to maximize variance. Resource limitations and programmatic priorities were considered when determining the margin of error (MOE) for each substrata. Since this study’s focus is private facilities we set the MOE ±5% for private facilities in the SHOPS Plus network. For public facilities the MOE is ±10%. This detail has been added to the manuscript (see line 218-225). Additional detail is also available in Annex S1.

*7. Line 254, please provide reasons with frequency for failed interactions*

a. We have added detail on complete and incomplete interactions. See Line 360-371. There were a total of 162 incomplete interactions (n=85 in Case 1, n=77 in Case 2). Reasons for incomplete interactions include: SP inability to find or initiate an interaction in a facility due to prolonged facility closure or provider absence after multiple attempts (n=80 in Case 1, n=27 in Case 2), the facility requested to re-test the SP for TB before proceeding with treatment initiation (n= 37 in Case 2 only), or the facility was no longer engaging with TB patients (n=1 in Case 1, n=4 in Case 2). In a small number of attempts SPs unexpectedly interacted with an individual identifying as a SHOPS Plus staff member (n=1 in the Case 1, n=5 in Case 2) or the SP noted that the provider had regarded them with suspicion or accused them faking their illness to obtain drugs on the black market (n=1 in Case 1, and n=4 in Case 2). All incomplete interactions were not included in analysis.S2 Annex provides further details on response rates.

*8. Table 3, be consistent with listing the reference category first or last (typically listed first)*

a. Thank you for making this observation. We’ve made this correction in Table 3, with the reference category listed first.

*9. line 320, I'd prefer to see CIs for proportions over SEs*

a. For reporting of significance testing between private and public DOTS facilities we have included CIs in the manuscript text. See lines 463-473 and 511-515.

*10. The authors may wish as a sensitivity analysis to calculate correct management according to the lenient definition used in other SP studies to allow direct comparison to these study results*

a. Most other TB-related SP studies have examined case management in clinical facilities and explored quality using a “textbook” presumptive TB SP scenario, using a lenient definition of correct management (i.e., providing or recommending an appropriate TB diagnostic). This definition corresponds directly to our correct management criterion on sputum sampling, so by directly comparing results for that sub-outcome we can see how the results from this study compare to other SP studies. Especially in comparison to other private clinical facilities, the results on sputum testing sub-outcome from Nigeria compare favorably. We’ve added further details on this in the Discussion, see lines 519-527 and added Annex S5 with further details.

Reviewer #2:

1. *The authors should better contextualize what the findings for the private sector facilities that received the SHOPS intervention might mean for other private sector facilities in Nigeria, including the following: (a) what kind of training did the SHOPS program provide to these private sector providers on TB care, including average number of educational contact hours; (b) to what extent do the facilities enrolled in the SHOPS program represent the “average” private sector facility in these geographic areas; and (c) describe any previous studies on TB care in the Nigerian private sector or at least what is known generally about the quality of private sector care in Nigeria. This is relevant because most studies with direct comparisons of public and private sectors in LMICs have generally shown better same or better quality of TB care in the public sector. See for example, the following systematic review that evaluated quality of TB care in India and compared public and private sector provider knowledge: Satyanarayana S et al. Int J TB Lung Dis 2015;19(7):751-763, or, a more relevant comparison, a SP study of TB quality of care in Kenya, which showed similar outcomes between public/private on most metrics but better for public in some: Daniels et al. BMJ Glob Health 2017;2(2):e000333. Therefore, the question is, how well does private sector care at SHOPS intervention sites represent private care at other sites?*
   a. Thank you for noting that more detail on the SHOPS Plus program and information on previous research on TB quality in Nigeria was missing from the manuscript. In Lines 73-82 we provide additional context on previous studies of quality in PPM pilots in Nigeria (e.g., all research to date has focused only on clinical providers and treatment success outcomes; though treatment success falls short of national goals, most of the studies that examined public and private providers together found comparable success rates). In Lines 136-196 we have added additional background information on the SHOPS Plus model, including how facilities were selected into networks and the training and support that they received. Considering that facilities participating in SHOPS Plus networks received fairly intensive support from SHOPS Plus and the facilities have to express willingness to participate in the TB program, network facilities do not represent average care at an unengaged private site. This study was designed for focus on and evaluate quality of care facilities engaged in a novel PPM model and was not intended to generalize to other settings.
2. *Need to place greater weight on particular key outcomes: Overall, I appreciate the authors’ use of bottleneck analyses as shown in the figures to represent the proportion who achieve all quality of care outcomes measured. With that said, some outcomes in these analyses are more important than others. For example, in case #1, undergoing a TB-related test (sputum, Xpert, CXR) is ultimately more important for diagnosis than the other metrics (e.g., asking appropriate questions about cough, etc.). Similarly, in case #2, treatment initiation is ultimately the most important outcome of that visit, compare to the other outcomes. For example, treatment initiation rate of 70% would be considered poor in many settings unless providers had an expectation that this would happen at the next visit. I think that the authors should mostly keep their current presentation of Results (the bottleneck analyses are excellent and informative), but also include a few lines that focus specifically on these key indicators and potentially conduct analyses that evaluate factors associated with completion or non-completion of these two key indicators for case #1 and case #2, since these two outcomes are so important. For example, it seems that, if the outcome of treatment initiation alone is looked at, private SHOPS clinical providers may outcome public sector providers (although they do not outperform on the composite quality of care outcome).*
   a. To understand whether and to what extent the private providers trained by SHOPS Plus in this study are in alignment with the major tenants of the Nigeria’s National TB program guidelines we’ve chosen to expand the definition of “correct” management beyond just sputum sampling as has been done in other SP studies to develop a more nuanced understanding of treatment. By clearly focusing on an overall measure of correct management, we can avoid confusion by casting some providers behaviors as more (or less) correct than others. Nevertheless, we do try to comment specifically on the “key” element of sputum sampling in Case 1 (see: lines 452-457, 522-527) and treatment initiation in Case 2 (see: Lines 479-486, 507-510, 601-604)

*3. For case #1, for the evaluation of whether providers provided unnecessary medications, fluoroquinolones, steroids, and TB medications (given without diagnostic workup) definitely count as unnecessary and potentially harmful for patients with presumptive TB as they might suppress symptoms and delay TB diagnosis (especially fluoroquinolones). However, whether narrow- or broad-spectrum antibiotics should count in this category of unnecessary drugs could be debated. For sputum smear-negative TB or Xpert-negative TB diagnosis, an empiric trial of (non-fluoroquinolone, non-TB) antibiotics is actually part of the diagnostic algorithm in most places, and it is a matter of clinical practice whether providers start these antibiotics while the initial TB workup is happening or not (it would not be unreasonable to do so depending on how sick you feel the patient is). I would recommend counting FQs, TB medications, and steroids as harmful and unnecessary drugs for this outcome, but potentially exclude other antibiotics.*

b. Thank you for this comment. While we agree that in practice it may be understandable for clinical providers to use their professional judgment and opt to commence treatment of a presumptive patient on broad-spectrum antibiotics while waiting for the results of a diagnostic test to come in, this practice is not in adherence with the diagnostic algorithm for management of presumptive TB that is defined in the NTBLCP’s guidelines (which is the standard by which we are judging “correct” management in this study). In this algorithm, a provider is to commence broad-spectrum antibiotics *after* confirming that the patient is either GX or AFB negative. Further, as we state in the manuscript on Line 291-297 there are important individual and public health justifications (e.g., preventing antibiotic resistance) for refraining from dispensing antibiotics in absence of a lab-confirmed diagnosis.

Nevertheless, given that we do expect providers to be motivated to provide services that they feel are in the best interest of their patients, we have run a sensitivity analysis in which we redefine the criterion on avoidance of prescription or dispensing of unnecessary or inappropriate medications to exclude broad-spectrum antibiotics as suggested. The original Case 1 results are robust to the sensitivity analysis. Although all cadre’s correct management increases by 3 or 4 percentage points this increase is not substantively different than when the more stringent criteria are applied, which implies that it is not dispensing/prescribing action that is driving whether providers correctly manage patients. See lines 401-407 also Annex S3 Tables D-G.

*4. Provider gender and outcomes: This was a fantastic part of the analysis, with women outperforming men in most cases. However, in the Results and Discussion, the authors did not comment on the fact that male providers seemed to also outperform in quality when their patients were women – whereas male providers performed more poorly when evaluating male patients. This is a potentially important finding. Analyses of prevalence survey data in comparison to notification data in many countries suggest that men with TB are under-diagnosed and/or under-notified and that there a considerable proportion of men never get diagnosed and started on TB treatment, see, for example: Horton KC et al. PLOS Medicine 2016; 13(9):e1002119. The authors should comment on this finding in the Results and Discussion.*

a. Your point about consistent under-diagnosis of TB in men globally and the fact that we do find evidence of men being managed less successfully than female patients in some circumstances does merit mention. We have now commented on this finding in the Discussion section. See Line 534-541. Thank you for this suggestion.

*5. The authors should be sure to provide the full expansion of every acronym as a foot note to every figure and/or table. For example, I found myself going back to look up what CP or PPMV stood for multiple times. Moreover, these acronyms themselves are confusing and the authors might consider using the full expansion for these terms in the text rather than the acronyms, especially since CP means “community pharmacy” but this is easy to confuse with “clinical providers.” In addition, it was not clear to me what the difference is between a CP and PPMV in the real world – they both seem to be private pharmacies, so the authors should describe how these two types of facilities are different in the background and setting section.*

a. Thank you for this suggestion. We’ve opted to reduce use of program or Nigeria-specific acronyms and refer to CPs as “pharmacies” and PPMVs as “drug shops” throughout. Thank you for this suggestion.

*2. In their description of the value of SP studies, instead of referring to this as a “gold standard” methodology, it might be safer to say that this is a high quality methodology for particular quality metrics. As the authors later note in the discussion, SP methods are only really good at measuring initial or single patient interactions and cannot measure subsequent quality of care well (e.g., during the TB treatment course, later in the diagnostic workup) – other methodologies would be higher quality for later steps.*

a. Thank you for making this observation. Although the literature we reference, including others who have implemented SP studies, refer to the SP methodology as a “gold standard” we think you make a fair point and agree with you about SP studies being suitable only for exploring quality at a single point in time. This has been noted already in the limitations, but we’ve also revised the language beginning on Line 202-203 to more accurately describe where SP studies are best deployed.

3. *Page 15, line 307: I believe the adjusted OR is reported incorrectly – should be 0.02 NOT 0.2.*

a. Thank you for correcting this error. It has been corrected in the revised manuscript. See Line 441.
4. *Page 16, lines 320-327: I think it is perhaps inappropriate to compare public clinical providers to other types of private sector sites—e.g., labs, pharmacies, etc. As you note, private labs have the job of trying to get people to do tests and so are not surprisingly more keen on getting patients to get tested. Instead, would restrict this public clinical providers comparison to the comparable group in the private sector—private clinical providers.*
a. Although we agree that labs (as well as other non-clinical cadres included in this study) operate differently than clinical facilities, in the context of the SHOPS Plus program, the non-clinical cadres were trained to manage presumptive patients in a similar manner as clinical providers in the private and public sectors. Since a goal of the SHOPS Plus program is to expand presumptive patient identification to other, non-clinical settings we chose to include all provider cadres in the program in the public sector comparison analysis. As we state in the manuscript the purpose of this comparison is to better understand how SHOPS Plus cadre are managing presumptive patients as compared to the pre-program status quo (which in this study is proxied by the public sector, see Line 343-347).
